# Selective parallel G-quadruplex recognition by a NIR-to-NIR two-photon squaraine†
†Electronic supplementary information (ESI) available: Synthesis, titration experiments, optical spectra, NMR and HRMS spectra. See DOI: 10.1039/c8sc02882f


**DOI:** 10.1039/c8sc02882f

**Published:** 2018-09-14

**Authors:** Vincenzo Grande, Chia-An Shen, Marco Deiana, Marta Dudek, Joanna Olesiak-Banska, Katarzyna Matczyszyn, Frank Würthner

**Affiliations:** a Universität Würzburg , Institut für Organische Chemie , Am Hubland , 97074 Würzburg , Germany . Email: wuerthner@uni-wuerzburg.de; b Center for Nanosystems Chemistry & Bavarian Polymer Institute (BPI) , Universität Würzburg , Theodor-Boveri-Weg , 97074 Würzburg , Germany; c Advanced Materials Engineering and Modelling Group , Faculty of Chemistry , Wroclaw University of Science and Technology , Wybrzeze Wyspianskiego 27 , 50-370 Wroclaw , Poland . Email: katarzyna.matczyszyn@pwr.edu.pl

## Abstract

Fluorescence imaging probes for specific G-quadruplex (G4) conformations are of considerable interest in biomedical research. Herein, we present the synthesis and the binding properties of a new water-soluble near-infrared (NIR) amphiphilic squaraine dye (**CAS-C1**) which is capable of selective detection of parallel over non-parallel and non G4 topologies. The striking changes in its linear optical response upon binding to parallel G4s give rise to high fluorescence quantum yields (*Φ*_f_ ≈ 0.7) and one-photon molecular brightness in the far-red-NIR region. The outstanding recognition process of **CAS-C1** for parallel G4s *via* end-stacking provides binding constants in the nanomolar regime (*K*_b_ = 10^7^ to 10^8^ M^–1^) awarding it as one of the most potent parallel G4 binders currently available. Moreover, the **CAS-C1**-parallel G4 system exhibits large two-photon absorption (TPA) cross-sections and molecular brightness in the second NIR biological transparency window (*λ* ≈ 1275 nm), making it an ideal candidate for NIR-to-NIR ultrasensitive two-photon procedures.

## Abstract 2

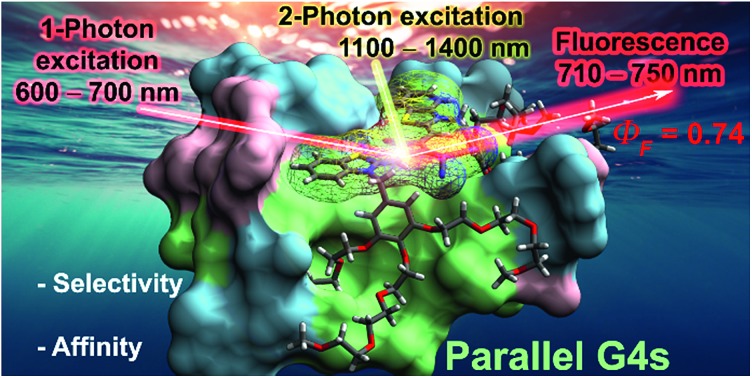
A selective and efficient nonlinear squaraine fluorescent probe for parallel G-quadruplexes suitable for NIR-to-NIR two-photon imaging procedures is reported.

## Introduction

**Fig. 1:**
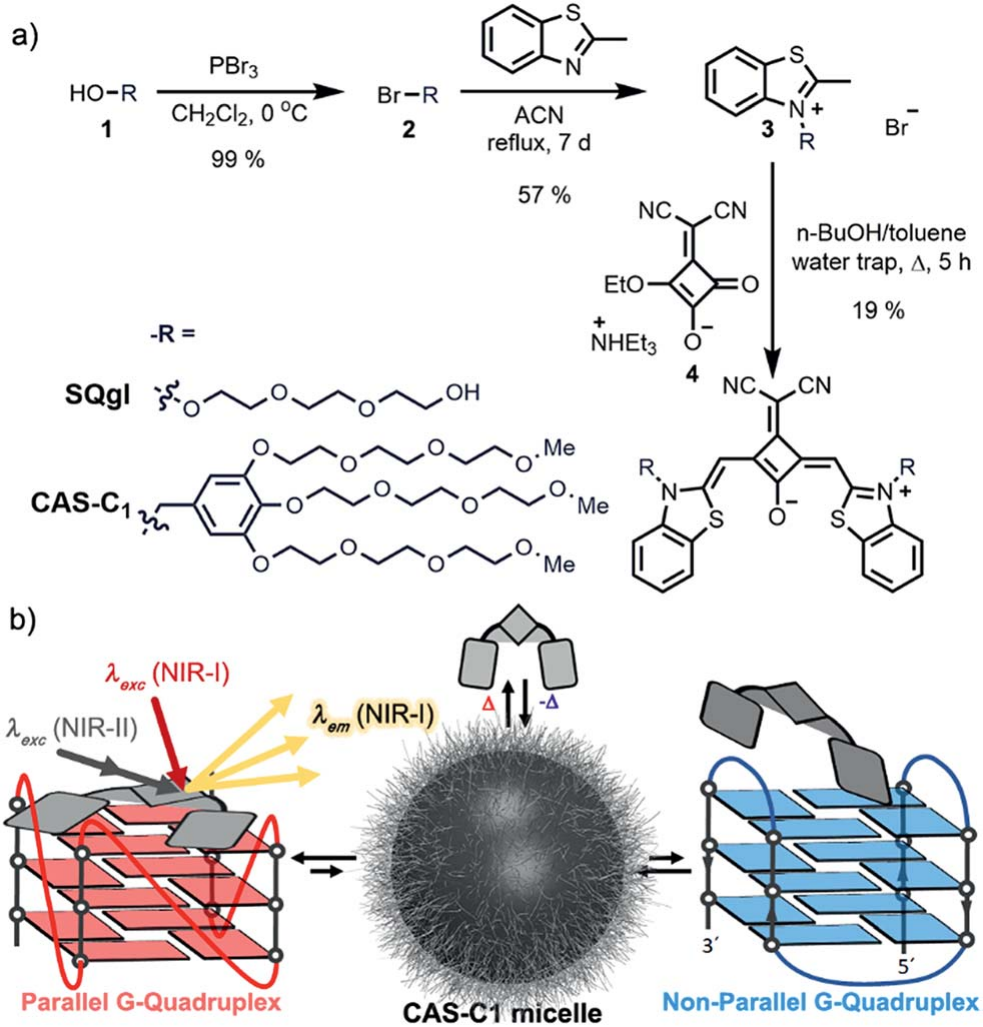
(a) Synthetic route to **CAS-C1**. (b) Schematic illustration of the multiphotonic selective detection mechanism: the squaraine shows amphiphilic assembly in water into micelles. The formation of a stable complex with parallel G-quadruplex (left) is able to displace the squaraine from the micellar aggregate and trigger the fluorescent emission in the NIR-I biological window upon both one-photon excitation in the NIR-I and two-photon excitation in the NIR-II. **CAS-C1** bestows high selectivity of interactions over non-quadruplex and non-parallel G4 structures (right).

Single-stranded guanine-rich oligonucleotides can fold into noncanonical compact, highly stable four-stranded helices known as G-quadruplexes (from here denoted as G4s).^1^ Recent studies have demonstrated the occurrence of G4s in human cells and their implication in several key biological events especially those that are disease- and aging-related.^2^,^3^ As a consequence, they are recognized as potential drug targets. To date, over 700 000 G4s have been identified across the human genome,^4^ especially located in the telomeres at the ends of chromosomes and in the promoter regions of oncogenes. Although indisputable progresses have been made over the past years in the design of G4-binders,^5^–^10^ the development of more complex multitasking compounds that combine the optimization of both optical performances (excitation and emission wavelengths > 600 nm and high brightness) and G4 structural selectivity/affinity (G4 over duplex binding and among the G4 topologies, Fig. 1) remains highly challenging.^11^–^13^ Indeed, the overwhelming majority of the small optical imaging probes for G4 detection are commonly photoexcited by relatively high-to-moderate energy light (330–600 nm)^14^,^15^ in order to generate the emission between the visible and far-red region of the electromagnetic spectrum. Such energies, in particular UV or blue, are in themselves deleterious to cells as they can irreversibly damage DNA and/or induce formation of cytotoxic reactive oxygen species.^16^ Furthermore, their excitation and emission wavelengths are confined outside the first and second near-infrared (NIR) biological transparency windows (BTWs, NIR-I: 700–950 nm and NIR-II: 1000–1350 nm)^17^ where absorption and scattering from tissues and oxy/deoxygenated blood is significant, limiting their relevance to 2-D cell cultures and precluding their use in more realistic diagnosis environments, such as in thick tissues or *in vivo* models. The lack of biocompatible fluorescent organic probes in the BTWs has prevented the use of these highly sensitive spectral ranges for in-depth imaging.^17^ In addition, the dynamic nature of the G4 structure enable a range of different topologies (*e.g.* parallel, hybrid and antiparallel) to potentially form under different physiological conditions, making molecular recognition particularly arduous. In our research on G4-probes, we have recently shown that a dicyanovinyl squaraine dye (**SQgI**, Fig. 1a)^18^ could behave as G4-specific NIR light-up probe. In the complexed state, **SQgl** is emissive at 700 nm upon excitation at 661 nm through one-photon absorption (OPA) with an impressive quantum yield of *Φ*_F_ ≈ 0.6. The probe exhibited good selectivity of G4 over duplex, with the dicyanovinyl squaraine dye capable to impart outstanding selectivity over non-G4 structures and remarkable specificity toward parallel G4 topologies. However, this probe presented inherent optical, practical and affinity limitations including: (i) moderate solubility, (ii) moderate binding constants (*K*_b_ values comprised between 10^5^ to 10^6^ M^–1^)^18^–^20^ and (iii) excitation wavelengths outside the BTWs. Thus, we speculated that exploring the chemical space around the squaraine core should enable optimization of both optical and DNA binding performances and we decided to decorate the dicyanovinylene squaraine moiety with bulkier water-soluble brush-type groups.

Herein, we present the first example of a water-soluble amphiphilic squaraine dye (hereafter **CAS-C1**, Fig. 1a) exhibiting G4-selective fluorescence light-up upon photoexcitation both within the NIR-I (*λ*_max_ ≈ 720 nm and *Φ*_F_ ≈ 0.7) through OPA, and within the NIR-II (two-photon absorption cross-section, *σ*_2_, of ≈ 300 GM and two-photon molecular brightness, *σ*_2_ × *Φ*_F_, of ≈ 200 GM at 1275 nm) through TPA. Moreover, the unprecedented nonlinear optical properties of **CAS-C1** are combined with the outstanding selectively for parallel over non-parallel G4 architectures and duplex morphologies of any length and composition awarding it as the best two-photon parallel G4-specific probe currently available.

## Results and discussion

### Design and synthesis

With the aim to increase the binding selectivity and the solubility of ligand **SQgl**, we decorated the dicyanovinylene squaraine core scaffold with brush substituents carrying six triethylene glycol chains (synthesis and characterization in ESI). The massive presence of oligoethylene glycol chains is not only beneficial for the solubility of the ligand, but it is also known to strengthen the affinity of the ligand toward the parallel G4s upon noncovalent interactions within the grooves. This approach is reported in the literature and has been exploited in the design of selective G4-ligands.^21^–^23^ The molecular design of dicyanovinyl squaraine revealed a suitable choice in terms of affinity and selectivity with G4s.^18^ Additionally, the donor–acceptor–donor (D–A–D) nature of the chromophores^24^ appeared very promising for multi-photon absorption processes at long-infrared wavelengths. The synthesis of the newly designed squaraine according to the route shown in Fig. 1a starts from the reported brush precursor **1** that was brominated to give the corresponding bromide **2**, which underwent a nucleophilic substitution with methyl-benzothiazole to give **3**. Following the reported synthetic procedure for dicyanovinyl squaraines,^24^**CAS-C1** was obtained after condensation of the squaric ester **4** with two equivalents of methyl-benzothiazolium **3**. Squaraine **CAS-C1** revealed fairly good solubility in various organic solvents. The absorption and emission spectra in these solvents revealed a moderate negative solvatochromism (Fig. S2†) and can be assigned to monomeric species (Fig. S1†).

### Aggregation in water

**Fig. 2:**
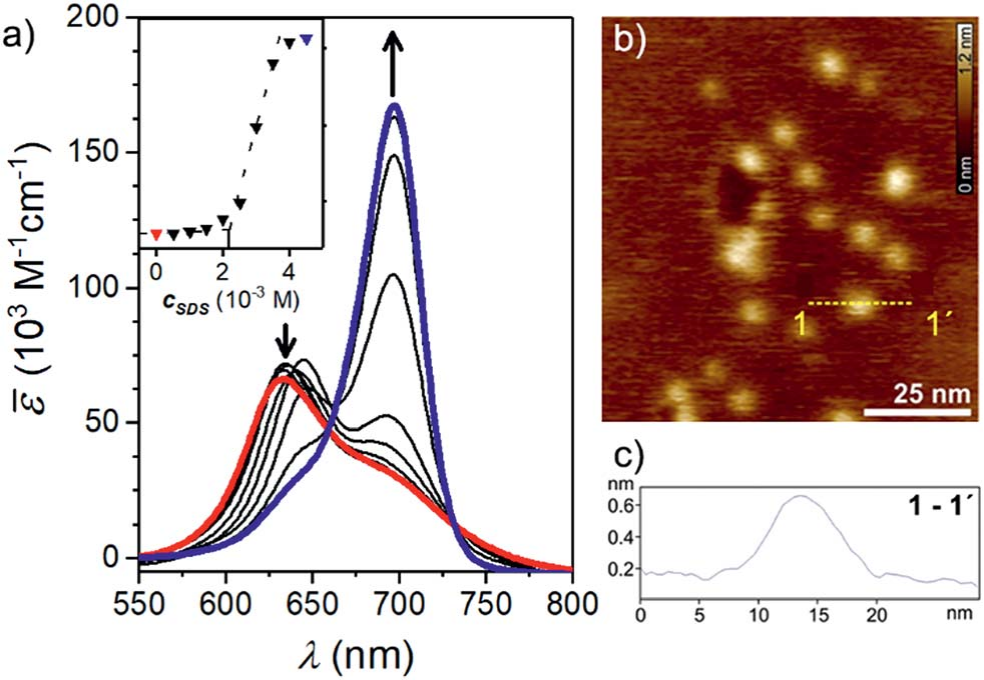
(a) UV/Vis absorption spectra of an aqueous **CAS-C1** solution (*c* = 3.0 × 10^–6^ M, 25 °C, red line) and upon addition of increasing amounts of SDS. The inset shows the incorporation of the squaraine dye in the SDS micelle upon addition of the surfactant with a critical concentration of 2.1 × 10^–3^ M. (b) AFM height images on mica after spin coating the **CAS-C1** aqueous solution (3.0 × 10^–6^ M). *Z* scale is 1.2 nm. (c) Cross-section from yellow dashed lines 1–1′.

In aqueous solution, **CAS-C1** absorption spectra reveal a different band shape and a strongly blue-shifted absorption maximum at 632 nm with an apparent extinction coefficient *φ̇*_max_ = 6.2 × 10^4^ M^–1^ (Fig. 2a, red line) which are first evidences for an H-type exciton coupling between aggregated dyes.^25^

Atomic force microscopy (AFM) revealed the presence of small particles with an irregular diameter of 10–30 nm and a height of 0.6–3.0 nm (Fig. 2b) which suggests the formation of micellar structures. The blue-shifted absorption band indicates a co-facial stacking of **CAS-C1** molecules, most likely in an antiparallel fashion in order to cancel the dyes' large dipole moment.^26^ Such arrangement explains well the strong H-type excitonic coupling and the quenched fluorescence emission. Due to the massive presence of ethylene glycol chains, **CAS-C1** is particularly suited to form such micellar structure where the hydrophilic parts are predominantly directed to the outside (Fig. 1b).

Upon heating to 95 °C, **CAS-C1** micellar aggregates become disfavored for entropic reasons leading to a new band at 676 nm with isosbestic point at 653 nm. These changes are reversible and are ascribed to the formation of the monomeric species in water, with the typical fluorescence emission peaked at 699 nm (Fig. S3A†). Global fitting of the temperature-dependent measurement in the range 550–750 nm is in good agreement with an isodesmic aggregation mechanism (Fig. S3B†). Despite the fact that the pure monomeric state is not fully reached in water, the extrapolation of the fitting at 676 nm predicts a reasonable value for monomeric **CAS-C1** of *φ̇*_max_ = 9.0 × 10^4^ M^–1^ cm^–1^. In accordance with the disaggregation model, a fluorescence emission in water lights-up at 699 nm (Fig. S3A†, dotted line). In contrast, the emission profile of **CAS-C1** in its aggregated form is fully quenched due to nonradiative relaxation pathways in its H-aggregate state. In order to investigate the photophysical properties of the excited states, time-correlated single photon counting (TCSPC) measurements of **CAS-C1** in water at 80 °C were performed. The time-dependent decay fitted better with the biexponential function (eqn S2, ESI†) suggesting the coexistence of two different species: one with longer lifetime (*τ*_1_ = 4.76 ns), consistently ascribed to the excited state of the free monomer in solution; the other one with shorter lifetime (*τ*_2_ = 1.35 ns), ascribed to the species aggregated in the micellar structure (Fig. S4†). The dramatic decrease of the lifetime and emission intensity is, therefore, in accordance with the formation of additional radiationless pathways in the aggregated form.

A more efficient approach to disassemble **CAS-C1** into monomeric dyes is provided by the addition of surfactant molecules. Thus, we performed titration with sodium dodecyl sulfate (SDS) (Fig. 2a). The surfactant affects moderately the formation of the micelles at lower SDS concentration. Above the critical concentration of 2.1 × 10^–3^ M, SDS induces a marked breakup of **CAS-C1** micelles by dissolving the **CAS-C1** monomer within the SDS micelles (Fig. 2a).

### G4 binding

**Fig. 3:**
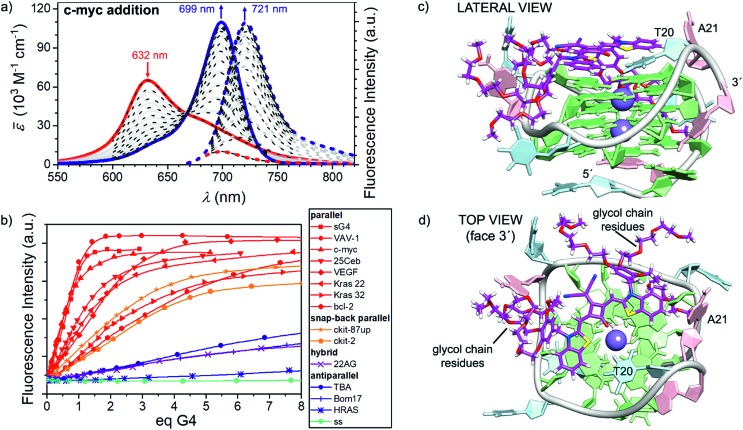
(a) Visible/NIR absorption (solid lines) and fluorescence spectra (dotted lines, excitation on the isosbestic point at 660 nm) of a **CAS-C1** buffered solution in water (1.66 × 10^–6^ M, TRIS buffer *c* = 10 mM, pH = 7.2, KCl *c* = 100 mM) upon addition of c-myc G4 at 25 °C (red and blue lines correspond to the spectra at 0 eq. and 3.4 eq. respectively. Superimposed black dotted line results from a global fitting with a 1 : 1 binding model (*K*_b_ = 9.7 × 10^6^ M^–1^). (b) Fluorescence titrations of the **CAS-C1** buffered solution in water upon addition of various G4s sequences at 25 °C: the plots show a clear-cut preference of the squaraine for the parallel G4s (in red). (c and d) Docking lowest energy binding models for **CAS-C1** with c-myc (score function Δ*E* =–5.9 kcal mol^–1^).

In a similar fashion to the disaggregation induced by SDS, molecular recognition of **CAS-C1** to suitable π-surfaces should also cause the disassembly of the micelle. This idea prompted us to study the recognition process of the squaraine toward a large panel of biologically relevant G4 topologies such as those found in the human telomeres (Tel-22) and minisatellite (Ceb25), in the proto-oncogene involved in the tumorigenesis of urinary bladder cancer (hRAS1), in the promoter region of myc, kit, KRAS, bcl2, and vav genes (c-myc, c-kit2, c-kit87up, KRAS 32, KRAS 22, bcl2 VAV1), in the vascular endothelial growth factor (VEGF) gene, in the telomeric transcripts (TERRA) and the thrombin-binding DNA aptamer (TBA) (see ESI† for details concerning the G4 topologies and their folding process). Indeed, upon addition of parallel G4s (*e.g.* VAV-1, c-myc and VEGF) the disappearance of the absorption band of **CAS-C1** aggregate along with the formation of a new bathochromically shifted band centered at ≈ 700 nm indicate the formation of G4 (parallel):**CAS-C1** complexes (Fig. 3, S6 and S7†). Concomitant with absorption changes, the steady-state emission of the squaraine fluorophore upon excitation at *λ*_exc_ = 660 nm, almost fully quenched in its aggregated unbound state (*λ*_em_ = 698 nm), lights-up in the NIR-I at *λ*_em_ ≈ 720 nm (Fig. 3a, S8 and S9†).

Notably, both the absorption and the emission bands are ≈ 20 nm redshifted compared to the spectrum of the monomeric squaraine in water (Fig. S3†). This is a clear indication of a direct interaction of the cyanine π-surface of **CAS-C1** with the guanines of the parallel quadruplexes. The excitation spectra and the lifetime decays recorded for **CAS-C1** in the presence of G4s indicate that the strong photoluminescence observed arises mainly from the **CAS-C1** : G4 complex through geometrical and structural restrictions which inhibit the nonradiative relaxation pathways, hence the increased lifetime and *Φ*_F_ (Fig. S10–S12†). TCSPC decay trace of the **CAS-C1** : G4 excited states (Fig. S1 and S12†) showed the presence of only one species, ascribed to the complexed squaraine, in accordance with the monoexponential function (eqn S1, ESI†). The singlet state lifetimes obtained by fitting of time-resolved fluorescence decays of the **CAS-C1** : G4 complexes are generally longer than the ones in organic solvents and comprised in the range of 5–6 ns (Fig. S2B†). This relatively long lifetimes with outstanding *Φ*_F_ in the 700–800 nm range makes **CAS-C1** particularly intriguing candidate as a fluorescence lifetime-based probe for G4 live-cell visualization by fluorescence lifetime imaging microscopy (FLIM).

Comparison of absorption and fluorescence responses between parallel and antiparallel G4s as well as single- and double-stranded DNA of any length and composition indicates a clear-cut binding preference of **CAS-C1** for G4 parallel topologies (Fig. 3b). This was further confirmed by duplex DNA competition titration assays (Fig. S19 and S20†) which revealed very modest interference of interaction with c-myc and VAV-1, even in the presence of 50 eq. of the *ds*-DNA.

It turned out that probe accommodation within the hydrophobic G4 scaffolds exert changes in the polarity of the surrounding that well-match a *E*_T_^*N*^ value of 0.6 (Fig. S2†). The optical changes arising in the absorption and emission profiles of the **CAS-C1**-parallel G4 systems result in excellent one-photon brightness (defined as the product *ε* × *Φ*_F_) in the NIR region with values comprised between 56 and 84 × 10^3^ M^–1^ cm^–1^ (Table 1).

**Table 1:** Summary of the spectroscopic properties and the binding constants of the complexes of **CAS-C1** with various G4s^*a*^

G-quadruplex	*λ*_max_ (nm)	*ε*_max_ (10^3^ M^–1^ cm^–1^)	*λ*_em_ (nm)	*K*_11_ (M^–1^)	*K*_12_ (M^–1^)	*τ* (ns)	*Φ*_F_	*ε*_max_ × *Φ*_F_ (10^3^ M^–1^ cm^–1^)
sG4	699	114	722	8.1 × 10^6^	—	5.8	0.74 ± 0.07	84
VAV-1	699	118	722	1.2 × 10^7^	—	5.6	0.67 ± 0.02	79
c-myc	699	108	721	1.0 × 10^7^	—	5.5	0.52 ± 0.03	56
25Ceb	698	108	719	9.0 × 10^5^	—	6.0	0.66 ± 0.01	71
VEGF	699	113	721	1.6 × 10^2^	3.7 × 10^9^	5.7	0.74 ± 0.08	84
Kras 22	699	116	721	1.5 × 10^7^	1.7 × 10^6^	5.4	—	—
Kras 32	699	103	721	5.9 × 10^6^	4.1 × 10^5^	5.6	—	—
bcl-2	699	113	721	36	6.0 × 10^8^	—	—	—
ckit-87up	699	103	721	3.1 × 10^2^	4.3 × 10^8^	5.5	—	—
ckit-2	699	92	720	5.0 × 10^2^	4.0 × 10^8^	—	—	—

^*a*^Data fitting with 1 : 1 and 1 : 2 models was performed according to ref. 28 and the software provided at the website http://supramolecular.org. We like to note that the credibility of the derived values for the 1 : 2 case is lower than for the 1 : 1 case. This is due to the presence of three species and unknown fluorescence quantum yields of the 1 : 1 and the 1 : 2 complexes, and changing amounts of these complexes during the titration experiment. To give the same weight to UV/Vis and fluorescence experiments we evaluated both titrations independently and took the average values.

As suggested by recent publications,^27^–^29^ the issue of the binding stoichiometry of the complexes in solution was assessed by Job's plot titration assays (Fig. S13–S15†), the tangent method and a global fitting based on both the 2 : 1, 1 : 1 and 1 : 2 binding models (Fig. S16–S18†). If compared with the previously reported **SQgl**, **CAS-C1** also showed multiple binding with the G4s, except for sG4, VAV-1 and c-myc.

A detailed quantitative binding data analysis that takes into account 1 : 1 and 1 : 2 **CAS-C1** : G4 stoichiometries with associated (anti-)cooperative effects indicates that **CAS-C1** exhibits higher binding constants for parallel topologies such as c-myc (Fig. 3c and d) with respect to antiparallel and non-G4 topologies with values that exceed by two-orders of magnitude those for the previously reported squaraine **SQgI** (see ESI† for details concerning the quantitative binding data analysis).^18^ These results are ascribed to the highly accessible parallel G-quartets which, being devoid of adjacent lateral or diagonal loops, provide better π-stacking platforms for the accommodation of the squaraine core, thereby resulting in a better planarity and thus in a more efficient rigidification as desired for a fluorescence increase. On the other hand, the negative effect of the external quartets surrounded by loops in antiparallel G4s such as 22AG and c-kit hampers to some extent the end-stacking ability of **CAS-C1** inducing a lower overall optical response. The aforementioned rationale is confirmed by using the closely related c-myc G4 analogue sG4, featuring no flanking overhang residues at both 5′ and 3′ ends that limit the steric hindrance, to enhance the light-up ability of **CAS-C1**. Indeed, complexation of the squaraine with sG4 provides a *Φ*_F_ of 0.74 which is among the highest values reported for nucleic acid complexes within comparable spectral range (Table 1).^30^,^31^ Structural insights into the binding mode of **CAS-C1** with G4s were provided by docking binding analysis on the G4s whose structures in solution had been derived by NMR studies under similar experimental conditions. The elucidated structures of G4s are referenced in Table S1†. In the case of c-myc, the squaraine chromophore is the recognition unit leading to a perfect complementary match with the 3′-quartet (Fig. 3c). However, the substitution of side groups at the squaraine chromophore has surely an impact in the binding. On one side, the massive number of glycol chains are responsible for the enhanced interaction compared to **SQgl** by establishing strong electrostatic interactions with the polar backbone of the G4. Presumably, the glycol chains displace water molecules from the grooves and thereby provide an entropic contribution to the stability of the G4-**CAS-C1** complex, especially for the parallel ones,^32^ which explains the strengthening of the binding affinity compared with **SQgl**. On the other hand, it is reasonable to assume that the steric hindrance of the benzyloxy unit, which is accommodated in the hydrophobic pockets in the side of the quartet (Fig. 3c and d), largely contribute to the selectivity of the interaction.

### Two-photon absorption

**Fig. 4:**
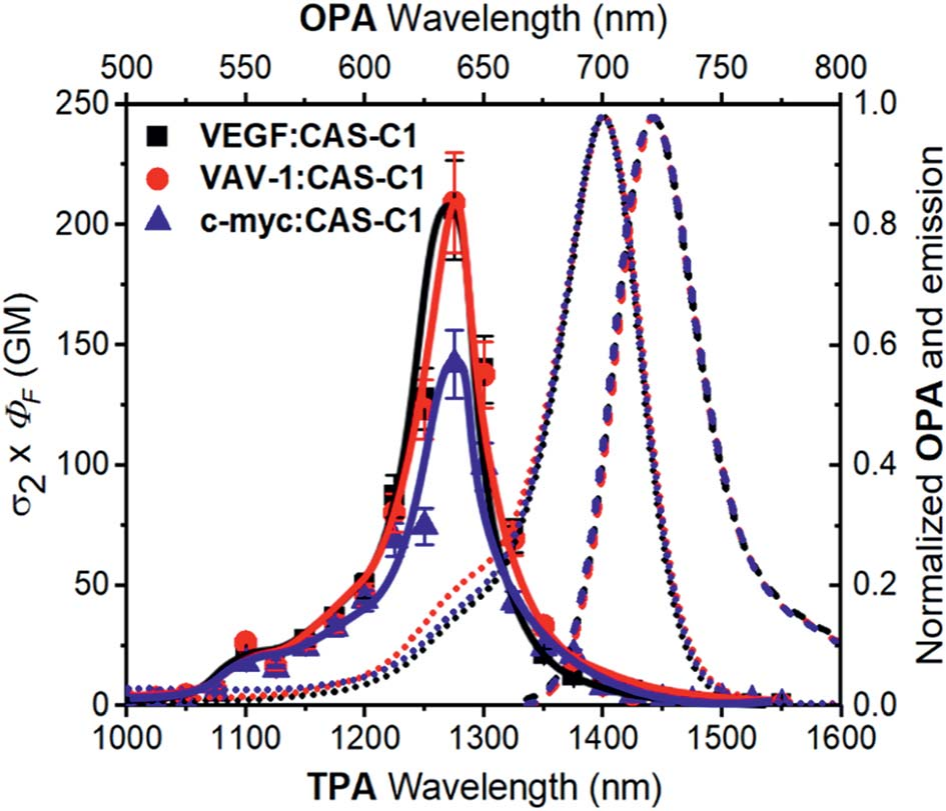
Two-photon molecular brightness (*σ*_2_ × *Φ*_F_) plot of **CAS-C1** in the presence of VEGF, VAV-1 and c-myc (10 mM Tris, 100 mM K^+^, pH 7.2) at molar ratio (*r*) = 7. The values of the *Φ*_F_ used are tabulated in Table 1. As a comparison, the OPA corresponding to the double of the energy of the TPA is shown (top and right axis): the absorption (dotted lines) and emission (dashed line) spectra corresponding to the **CAS-C1** complexes with the G4s are shown.

A fundamental optical limitation is encountered at the nanoscale level owing to Abbe's law of diffraction, which limits the maximum spatial resolution to approximately half the wavelength of light used to image the specimen.^33^ One way to overcome this limitation involves the use of nonlinear optical procedures based on multi-photon absorption processes. In this approach, the excitation of a chromophore is performed by the simultaneous absorption of two-photons with half the energy required for the corresponding one-photon absorption process. As a result, the excitation wavelength is confined in the first and second NIR biological transparency windows where the diffusion and absorption of light by inherently biological matrices is minimized, and thus allowing deep tissue penetration. Moreover, the low-energy excitation prevents photobleaching and cellular damages in biological samples. Importantly, the TPA is limited to a small femtoliter volume and occurs only at the focal point of a focused laser beam resulting in 3D image resolution. Despite the lower energy of the IR light compared to visible and UV light, this excitation mode has been proven to induce damage to the samples when using classical fluorophore that possess low TPA cross-section (*σ*_2_ < 50 GM).^34^ It turns out that, besides having significant photoluminescence properties in the NIR, a chromophore should also possess high *σ*_2_ values to be considered as a suitable probe for two-photon microscopy procedures in NIR-to-NIR configurations. The TPA spectra of **CAS-C1** complexed with parallel G4s including VAV1, VEGF and c-myc were measured by two-photon excited fluorescence (TPEF) method in the range 1000–1550 nm and are reported in Fig. 4 as *σ*_2_ × *Φ*_F_.

In general, all the complexes show similar TPA spectra composed of an intense high energy TPA band blue shifted compared to the one-photon transition energy and a weak low energy TPA band that matches well the wavelength-doubled 1PA spectra. These spectral features are usually associated with centrosymmetric quadrupolar molecules, in which selection rules for 1PA and 2PA electronic transitions are mutually exclusive. **CAS-C1** presents a non-centrosymmetric character with a quasi-*C*_2v_ symmetry and can be treated as bent quadrupolar chromophore, of which the quadrupolar conformation gives rise to an intense hypsochromically shifted TPA while the residual dipolar character results in a weak TPA transition overlapped with the 1PA maximum. Similar conclusions were recently reported for extended Michler's ketone, heptamethine, diarylboryl and anthracenyl derivatives.^35^–^38^ The TPA cross-section (*σ*_2_) values for the **CAS-C1** : G4 systems follow the order: **CAS-C1** : VAV1 *σ*_2_ = 312 GM, **CAS-C1** : VEGF *σ*_2_ = 278 GM and **CAS-C1** : c-myc *σ*_2_ = 273 GM at 1275 nm (Fig. S25†). Interestingly, the **CAS-C1** aggregate also shows TPA absorption close to half energy of the corresponding OPA, in agreement with a centrosymmetric aggregate (Fig. S26†).

The high values of *σ*_2_ combined with the associated 2PA maximum are an optimal combination for NIR-to-NIR configurations since excitation of **CAS-C1** : G4 systems can be performed within the NIR-II window by TPA (*λ*_ex(2PA)_ = 1275 nm) with emissions centered within the NIR-I spectral region (*λ*_em_ ≈ 720 nm). In line with the trend depicted for *σ*_2_ also the two-photon molecular brightness (*σ*_2_ × *Φ*_F_), as a result of the strong fluorescence enhancement of **CAS-C1** bound to parallel G4s, follow the same order (*i.e.***CAS-C1** : VAV1 *σ*_2_ × *Φ*_F_ = 209 GM, **CAS-C1** : VEGF *σ*_2_ × *Φ*_F_ = 206 GM and **CAS-C1** : c-myc *σ*_2_ × *Φ*_F_ = 142 GM at 1275 nm) indicating the suitability of **CAS-C1** to be used as a nonlinear biomarker for parallel G4 detection by using ultrasensitive fluorescence microscopy techniques. These values are nevertheless lower than those observed for monomeric **CAS-C1** in organic solvent (*σ*_2_ × *Φ*_F_ = 386 GM in isopropanol, Fig. S24†). This result is in agreement with recent results from Goodson and co-workers^39^ and some of us^40^,^41^ that relate the changes in the TPA cross-section to the different DNA binding modes. In particular, groove binders exhibit an enhanced TPA cross-section due to the DNA electric field induced enhancement of the transition dipole moment, while intercalators or end-stackers show a decrease in the TPA cross-section when complexed with DNA. Accordingly, this finding also supports our proposal that **CAS-C1** binds to parallel G4s mainly *via* end-stacking mode in agreement with the docking results. The end-stacking ability of **CAS-C1** was further confirmed through electronic circular dichroism (ECD) measurements (Fig S27†).^42^ Indeed, the native conformation of the parallel G4s was almost not affected by the presence of the squaraine dye indicating no or very weak perturbation on the quadruplex morphologies. By analogy with previously reported data for a G4 intercalator,^41^ the absence of conformational changes observed for **CAS-C1**-parallel G4 systems constitutes a clear evidence of the ability of **CAS-C1** to coordinate the external G-tetrads, ruling out the possible intercalative binding behavior of our probe known to give rise to pronounced structural changes associated with a partially disruption of the stacking interactions that govern G4 formation.

## Conclusions

In conclusion, the herein reported results on a new squaraine dye **CAS-C1** with outstanding binding strength and selectivity towards parallel G4 motif open up a new research line for this fascinating class of NIR fluorophores and functional dyes^17^,^43^ beyond previous applications in organic electronics,^44^ photovoltaics^45^ and exciton transport.^46^ The **CAS-C1** probe once complexed with parallel G4s exhibits a NIR light-up fluorescence response with quantum yields that go beyond 0.7. As a result, close-to-optimal one-photon molecular brightness in the far red-NIR region can be obtained. The parallel G4 preference of **CAS-C1** is accompanied by binding constants that exceed by two-orders of magnitude those calculated for the previously reported squaraine **SQgI**. The selectivity of the probe is tested toward a large panel of anti-parallel G4s and single- and double-stranded morphologies of any size and composition making **CAS-C1** a highly discriminatory parallel G4-binder. The docking results showed that the squaraine chromophore bestows the necessary selectivity for the interactions with the G4s. The molecular design of **CAS-C1** allowed us, on the one hand, to increase the selectivity of the interaction by exploiting the more easily accessible G-quartet surface of parallel G4s and, on the other hand, the massive presence of glycol chains in the hydrophobic pocket of the G4 imparted strong affinity. The complexes of **CAS-C1** with parallel G4s also possess strong two-photon absorption with maxima centered at 1275 nm, which make them ideally suited for NIR-to-NIR imaging procedures. Ultimately, the enhanced fluorescence quantum yields and the two-photon absorption cross-section enable one to obtain exceptionally high two-photon molecular brightness values within the NIR-II window that are desired for virtually all ultrasensitive fluorescence spectroscopy and microscopy techniques. These overall findings award **CAS-C1** as the best two-photon parallel G4-specific probe currently available.

## Conflicts of interest

There are no conflicts to declare.

## Supplementary Material

Supplementary information
