# Supplementary material for: Selective parallel G-quadruplex recognition by a NIR-to-NIR two-photon squaraine
Source: Chem Sci. 2018 Sep 14;9(44):8375–81. doi: 10.1039/c8sc02882f (PMC6240894; doi:10.1039/c8sc02882f)
Supplement: Supplementary file 1 [file SC-009-C8SC02882F-s001.pdf]

Electronic Supplementary Material (ESI) for Chemical Science.
This journal is © The Royal Society of Chemistry 2018

Electronic Supplementary Information

Selective parallel G-quadruplex recognition by a NIR-to-NIR two-photon squaraine

Vincenzo Grande,[§ab] Chia-An Shen,[§a] Marco Deiana,[c] Marta Dudek,[c] Joanna Olesiak-Banska,[c] Katarzyna Matczyszyn*[c] Frank Würthner*[ab]

[a]Universität Würzburg, Institut für Organische Chemie, Am Hubland, 97074 Würzburg (Germany). E-mail: wuerthner@uni-wuerzburg.de.
[b]Center for Nanosystems Chemistry & Bavarian Polymer Institute (BPI), Universität Würzburg, Theodor-Boveri-Weg, 97074 Würzburg (Germany).
[c]Advanced Materials Engineering and Modelling Group, Faculty of Chemistry, Wroclaw University of Science and Technology, Wybrzeze Wyspianskiego 27, 50-370 Wroclaw, (Poland). E-mail: katarzyna.matczyszyn@pwr.edu.pl

Table of Contents

1. Materials and methods 2
2. Synthesis and characterization 6
   Synthesis of 2 6
   Synthesis of 3 7
   Synthesis of CAS-C1 7
3. Optical studies in organic solvents 9
4. Optical studies in aqueous media 10
5. G-quadruplex studies 11
   Kinetics 11
   G-quadruplex titrations 12
   Photophysical characterization 16
   Fluorescence lifetimes 17
   Job´s plot and stoichiometry 19
   Competition titrations 25
   Speciation analysis 27
   Two-photon absorption 30
   ECD Spectra 31
6. NMR and HRMS spectra of CAS-C1 32
7. References 34

1. Materials and methods

Solvents and reagents were purchased from commercial suppliers (Sigma-Aldrich, ACROS, Alfa Aesar and Merck) and used as received, unless otherwise stated. All reactions were carried out under nitrogen atmosphere. Column chromatography was performed using silica gel 60M (0.04-0.063 mm). Oligonucleotides were purchased from Sigma Aldrich, diluted with water to a concentration of 2.00×10^{-3} M and stored at -15 °C. For all the experiments involving oligonucleotides, DNAse free water and transferring equipment were used. Ultrapure water type 1 was obtained by using a Millipore Simplicity® System (13.1 MΩ cm). A stock solution of 1.00 M KCl in water was prepared using solid KCl. The aqueous stock solution (1.00 M) of TRIS buffer was prepared by dissolving tris(hydroxymethyl)aminomethane in water and the pH was then adjusted to 7.42 ± 0.02 by adding KOH.

^{1}H and ^{13}C NMR spectra were generally recorded on a Bruker Avance III HD spectrometer operating at 400 MHz (^{1}H) or 100 MHz (^{13}C), with the residual protic solvent used as the internal standard. The chemical shifts are reported in parts per million (ppm). Multiplicities for proton signals are abbreviated as s, d, and m for singlet, doublet and multiplet, respectively.

High resolution mass spectra (HRMS) were recorded on an ESI micrOTOF focus spectrometer (Bruker Daltonic GmbH, Germany) and the matrix-assisted laser desorption/ionization (MALDI) spectra were obtained with a Bruker autoflex II spectrometer.

Optical spectroscopy. For all optical measurements spectroscopic grade solvents (Uvasol®) and ultra-pure water were used. Jasco V-770 UV-Vis-NIR spectrophotometer was used with spectral band width and the scan rate of 1 nm and 240 nm min^{-1}, respectively. For aqueous solution temperature-dependent absorption measurements, the concentration was corrected for the temperature-dependent density change by using the experimental equation for water ($d = 1.59 \times 10^{-3}$ T^{3}-5.97×10^{-6} T^{2}+2.27×10^{-5} T+0.99994, where T is the temperature in °C). Fluorescence spectra were recorded on PTI QM-4/2003 spectrometer with additional NIR add-on kit. Fluorescence quantum yields were determined by optical dilution method ($OD_{max} < 0.1$) as the average value of four different excitation wavelengths (630, 640, 650 and 660 nm) using Rhodamine 800 (λ_{max} = 681 nm, λ_{em} = 698 nm in ethanol, Φ_F = 0.25 ± 0.03)[1] as reference. The signals were corrected for the different refractive indexes, according to the following equation:[2]

$$\Phi_F = \Phi_F^{ref} \times \frac{I_\lambda}{I_\lambda^{ref}} \frac{1 - 10^{-A_\lambda^{ref}}}{1 - 10^{-A_\lambda}} \frac{n^2}{n_{ref}^2}$$

where I_λ is the fluorescence intensity area in the range 667 – 850 nm. The slits width of both monochromators were set fixed at 5.0 nm.[3]

The UV/Vis measurements were performed in conventional quartz cell cuvettes with path lengths of 0.01 – 50.0 mm.

ECD spectra were measured with a Jasco J-815 spectropolarimeter (JascoInc, USA) equipped with the JascoPeltier-type temperature controller (CDF-426S/15) and are presented as a sum of 3 accumulations. ECD measurements were performed at 25 °C in the wavelength range of 210-310 nm at different **CAS-C1** / G4 ratios. Before use, the optical chamber of the CD spectrometer was deoxygenated with dry nitrogen and was held under nitrogen atmosphere during the measurements. Appropriate references were subtracted from the obtained CD spectra.

TCSPC and singlet lifetime. The buffered solution of squaraine and G4 was prepared as for the titration studies. The suitable amount of G4 was added in order to obtain a complete formation of the complex. The formation of the complex was monitored by UV/Vis spectroscopy, then emission and excitation spectra were recorded. Lifetimes were obtained by monitoring the decay in the emission maximum upon irradiation of the pulsed laser at 670 nm (laser pulse 100 ns, emission slit 6.0 nm).

Data were fitted with a monoexponential function (Eq. S1) or a biexponential function (Eq. S2):

$$I = A + B_1 e^{-\frac{t}{\tau_1}} \qquad \text{(Eq. S1)}$$

$$I = A + B_1 e^{-\frac{t}{\tau_1}} + B_2 e^{-\frac{t}{\tau_2}} \qquad \text{(Eq. S2)}$$

Atomic Force Microscopy (AFM). AFM measurements were performed under ambient conditions using a Bruker Multimode 8 SPM system operating in tapping mode in air. Silica cantilevers (OMCL-AC160TS, Olympus) with a resonance frequency of ≈ 300 kHz and a spring constant of ≈ 40 Nm^{-1} were used. The samples were prepared by spin-coating a 10 × 10^{-6} M **CAS-C1** aqueous solution onto mica with 2000 – 4000 rpm.

Oligonucleotide annealing. A suitable amount of 2.00 × 10^{-3} M G4 stock solution was diluted with buffered water (c_{KCl} = 100 mM**,** tris(hydroxymethyl)aminomethane TRIS buffer 10.0 mM, pH= 7.4), heated up at 95 °C for 10 min and then let it slowly cooled down to room temperature in nearly 3 hours. For UV/Vis titrations 500 µM solutions were used. The G4s used in these experiments, their sequence and topology is described in Table S1.

Table S1. Oligonucleotides utilized in this study purchased from Sigma Aldrich.

Name	Sequence[a]	Length	Topology and description
**KRAS 32**	AGGGCGGTGTGGGAAGAGGGAAGAGGGGGAGG	32	parallel[4]
**KRAS 22**	AGGGCGGTGTGGGAATAGGGAA	22	parallel[5]
**sG4**	GGGTGGGTAGGGTGGG	16	parallel
**bcl2**	GGGCGGGCGCGGGAGGAAGGGGGCGGG	27	parallel[6]
**25CEB**	AGGGTGGGTGTAAGTGTGGGTGGGT	25	parallel[7]
**c-myc**	TGAGGGTGGGTAGGGTGGGTAA	22	parallel[4]
**VAV1**	GGGCAGGGAGGGAACTGGG	19	parallel[8]
**VEGF**	GGGAGGGTTGGGGTGGG	17	parallel[6]
**TERRA**	AGGGUUAGGGUUAGGGUUAGGG	22	parallel[9]
**c-kit2**	CCCGGGCGGGCGCGAGGGAGGGGAGG	26	parallel (snap-back)[4]
**c-kit87up**	AGGGAGGGCGCTGGGAGGAGGG	22	parallel (snap-back)[10]
**22AG (A-Tel21, wtTel22)**	AGGGTTAGGGTTAGGGTTAGGG	22	hybrid/anti-parallel[11]
**hRAS1**	TCGGGTTGCGGGCGCAGGGCACGGGCG	27	anti-parallel[8]
**Bom17**	GGTTAGGTTAGGTTAGG	17	anti-parallel[7]
**TBA 15**	GGTTGGTGTGGTTGG	15	anti-parallel[6]
**ss SCR**	GGATGTGAGTGTGAGTGTGAGG	22	single strand[4]
**ds26**	CAATCGGATCGAATTCGATCCGATTG	26	duplex[4]

[a] Conventional 5' to 3' direction.

UV/Vis and fluorescence G4 titration. Aqueous solutions of squaraine **CAS-C1** were prepared by dissolving the compound in ultra-pure water and adding KCl and TRIS stock solutions (c_{KCl} = 100 mM, c_{TRIS} = 10.0 mM). The pH was checked by a Mettler Toledo Fivego® portable pH meter and adjusted to pH = 7.42 ± 0.04. The freshly prepared **CAS-C1** solution was titrated with the freshly annealed oligonucleotide buffered solution (500 µM). The titration experiments were followed by UV/Vis (UV/Vis bandwidth 2.0 nm, response 0.06 s, interval 0.5 nm, scan speed 400 nm/min) and fluorescence measurements (excitation at 660 nm, slit 4.00 nm, excitation polarizer at 0 °, emission polarizer at 54.8 °, iris setting 100, averaged on 3 repetitions) after each addition and nearly 25 min stirring in the dark (400 rpm, 25 °C). The concentration of the experiment was optimized to have *OD* < 0.20 to avoid reabsorption in the fluorescence emission.

Two-photon excited fluorescence studies. Two-photon absorption was determined from two-photon excited fluorescence (TPEF) spectra measured with a femtosecond laser system, which consisted of Ti:Sapphire laser Chameleon (Coherent Inc.) and an optical parametric oscillator Chameleon OPO (Coherent, Inc.) with an output signal tunable in the range 1000 – 1600 nm. The system produces pulses of the repetition rate equal 80 MHz and the pulse duration ≈ 200 fs. An output power was set to 100-120 mW for all the wavelengths, the same for each sample and a corresponding reference solution. The sample solution contained in a cuvette was illuminated with a microscope objective (Nikon Plan Fluor, 40x, NA 0.75) and collected in an epi-fluorescence mode. TPEF spectra were measured with Shamrock 303i

spectrometer (Andor) equipped with iDus camera (Andor). The TPA spectra were obtained by tuning the wavelength of OPO and measuring the corresponding intensity of TPEF. The absolute 2PA cross-sections were calculated using relative fluorescence technique,[12] where TPEF of the sample is compared with the signal of a standard under the same experimental conditions. We used styryl 9M as a standard.[13]

Data processing. All the data were corrected for the dilution upon titration with the G4 stock solution (500 μM). Binding constants were obtained with Bindfit (available on the website http://supramolecular.org/) by using multiple global fitting methods (Nelder-Mead method) of both the UV/Vis data in the range of 600 – 740 nm and fluorescence data in the range 690 – 764 nm. Dilution corrections option was included in the fitting option, as described in the references.[14,15] In order to ensure to find the minima in the fitting analyses, all the fittings were confirmed with three different start values.

Molecular modelling. Structural optimization of **CAS-C1** was carried out on its methylated precursor by Density Functional Theory (DFT) utilizing Gaussian09[16] suite of programs. The monomeric forms were optimized using B3LYP/6-311+G** and the solvent effect (THF, unless specified) was taken into account as PCM. Excited state was modelled using TD-DFT method.

For molecular docking, the coordinates of the structures were retrieved from the Protein Data Bank.[17] All structures were inferred from NMR studies in solution, under similar conditions (concentration, ionic strength, pH and temperature) of the experimental ones.

Docking studies were carried out with the Lamarckian genetic algorithm following the procedure developed for G-quadruplex DNA/RNA and ligand locking using Auto-dock Vina v1.1.2.[18] The dimensions of the active site box, that was placed always at the centre of the G-quadruplex, were set to 80 Å x 80 Å x 80 Å, with a grid space of 0.375 Å. **CAS-C1** was optimized before using Gaussian suite of program (B3LYP/6-31+G** level, in water modelled as polarizable continuum model with UAHF atomic radii). Two rounds of simulation were performed. In the first round, simulated annealing was used to find a rough binding mode of **CAS-C1** with 50 runs while keeping all other parameters default. The search space was subsequently reduced and another 200 runs were conducted to get a more precise result. Following the docking studies of **CAS-C1** with G-quadruplexes DNA/RNA, molecular graphics and analyses were performed with the UCSF Chimera package. Chimera is developed by the Resource for Biocomputing, Visualization, and Informatics at the University of California, San Francisco (supported by NIGMS P41-GM103311).[19]

2. Synthesis and characterization

Squaraine **CAS-C1** was synthesized according to the route depicted in Scheme S1. The staring compounds **1**[20] and **5**[21] were synthesized by literature-known procedures and 2-methylbenzothiazole **3** was obtained from commercial supplier.

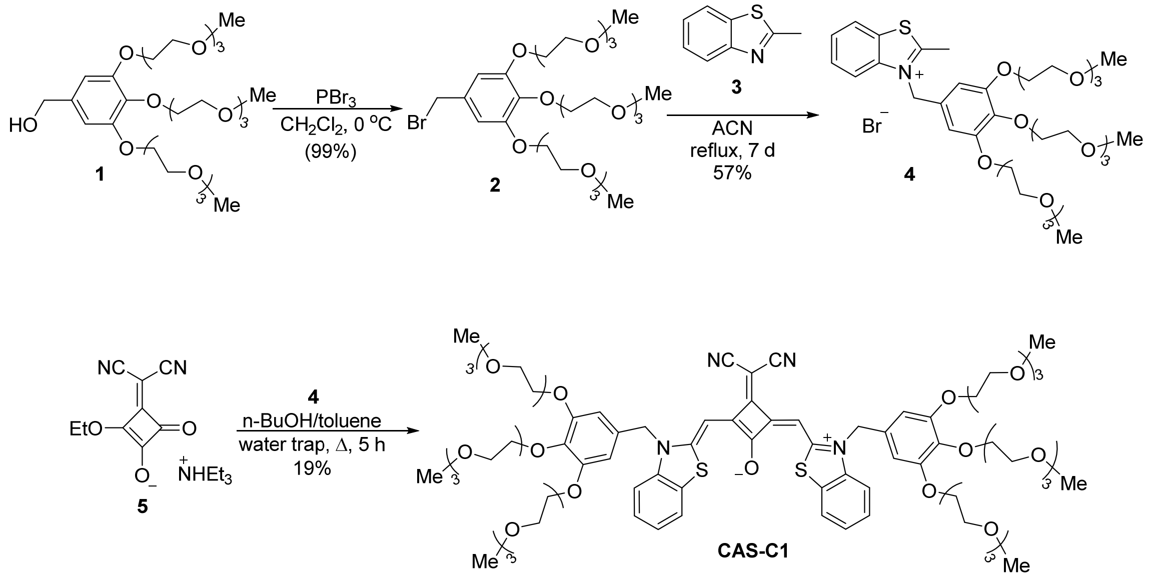

Scheme S1 Synthetic route to CAS-C1.

Synthesis of 2

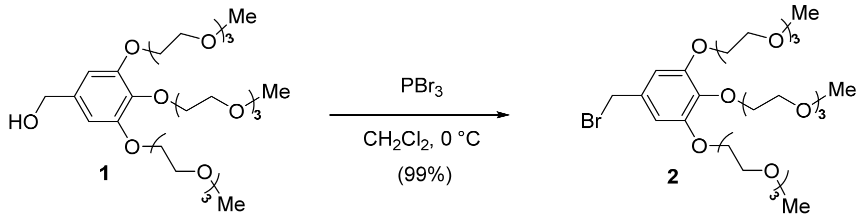

To a solution of benzyl alcohol derivative **1** (1.03 g, 1.73 mmol) in anhydrous dichloromethane (5 mL) PBr_3 (0.086 mL, 0.905 mmol) was added at 0 °C under nitrogen atmosphere. The reaction mixture was stirred for 2 h at 0 °C. Afterwards, water (50 mL) was added and the organic layer was separated. The aqueous phase was extracted with dichloromethane (100 mL × 3). The combined organic layer was washed with brine and dried over sodium sulfate. The solvent was removed under reduced pressure to obtain benzyl bromide **2** as a light brown liquid in nearly quantitative yield (1.14 g, 99%).

^{1}H NMR (400 MHz, $CDCl_3$): δ = 6.62 (s, 2H), 4.40 (s, 2H), 4.12-4.16 (m, 6H), 3.84 (t, *J* = 5.2 Hz, 4H), 3.77 (t, *J* = 5.0 Hz, 2H), 3.70-3.73 (m, 6H), 3.64-3.67 (m, 12H), 3.52-3.55 (m, 6H), 3.38 (s, 9H); ^{13}C NMR (100 MHz, $CDCl_3$): δ = 152.5, 138.5, 132.9, 108.6, 70.7, 70.65, 70.63, 70.5, 70.49, 70.47, 70.45, 69.6, 68.8, 59.01, 58.9,

34.0. MS (MALDI, pos. mode, matrix: DCTB 1:3, $CHCl_3$): *m/z*: 679.228 $[M+Na]^+$ (calcd for $C_{28}H_{49}BrNaO_{12}$ 679.231).

Synthesis of 3

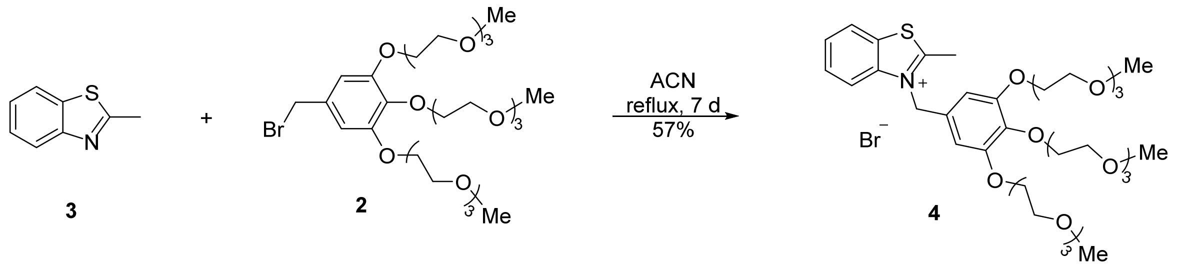

2-Methylbenzothiazole **3** (50 µL, 392 µmol) and **2** (1.03 g, 1.57 mmol) were mixed in 5 mL anhydrous acetonitrile (ACN) and heated to reflux for seven days under nitrogen atmosphere. The reaction mixture was then cooled down to room temperature and the solvent was removed under reduced pressure. The residue was dissolved in methanol (1 mL) and a large amount of diethyl ether (100 mL) was added to induce precipitation. The precipitate was collected by filtration, washed with diethyl ether, and dried under high vacuum to yield compound **4** as a viscous brown oil (182 mg, 58%).

^1H NMR (400 MHz, MeOD): δ = 8.34 (d, J = 8.2 Hz, 1H, H_{arom}), 8.21(d, J = 8.0 Hz, 1H, H_{arom}), 7.88 (td, J = 7.9 Hz, 1.2 Hz, 1H, H_{arom}), 7.82 (td, J = 7.9 Hz, 1.2 Hz, 1H, H_{arom}), 6.60(s, 2H, H_{arom}), 5.98 (s, 2H, N-CH_2), 4.10 (m, 6H, CH_2), 3.77 (m, 6H, CH_2), 3.63 (m, 18H, CH_2), 3.52 (m, 6H, CH_2), 3.34 (s, 3H, O-CH_3), 3.33 (s, 6H, O-CH_3), 3.29 (s, 3H, CH_3) ppm. ^{13}C NMR (100 MHz, MeOD): δ = 154.8, 142.9, 131.2, 129.9, 125.5, 118.3, 107.8, 73.7, 73.5, 73.0, 72.9, 71.8, 71.7, 71.6, 71.5, 71.4, 71.3, 70.8, 70.3, 62.2, 59.1, 53.7, 17.6 ppm. HRMS (ESI, pos. mode, MeOH): *m/z*: 726.3526 $[M^+]$ (calcd for $C_{36}H_{56}NO_{12}S$ 726.3518).

Synthesis of CAS-C1

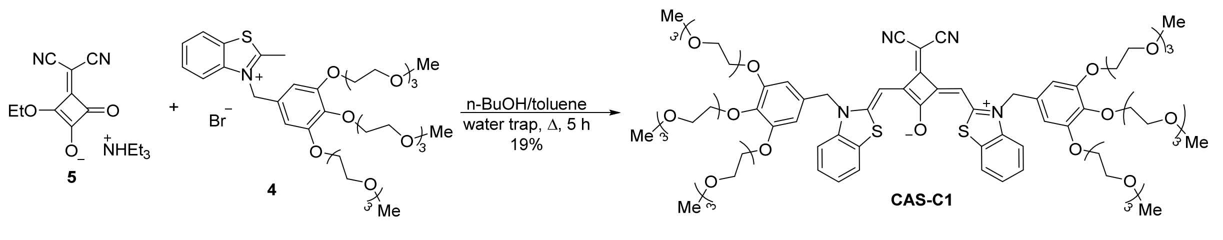

A round-bottom flux, equipped with a water-removing Dean-Stark apparatus, was charged with 4 mL *n*-butanol and 4 mL toluene. Subsequently, dicyanomethylene squaric ester **5** (13.0 mg, 44.6 µmol) and compound **3** (168 mg, 208 µmol) were added and the mixture was heated to reflux at 180 °C for 5 h. After being cooled down to room temperature, solvent was removed under reduced pressure. The residue was dissolved in CH_2Cl_2/MeOH = 4/1 solvent mixture (3 mL) and added dropwise into 500 mL of pre-cooled diethyl ether. After 1 h of vigorous stirring, the precipitate was collected by filtration and washed with

diethyl ether. The crude product was further purified by flash chromatography ($CHCl_3$/MeOH = 97/3) to yield a viscous green solid (13.8 mg, 20%).

^{1}H NMR (400 MHz, $CDCl_3$): δ = 7.59 (dd, J = 7.8, 0.6 Hz, 2H, H_{arom}), 7.42 (td, J = 7.8 Hz, 1.1Hz, 2H, H_{arom}), 7.30 (m, 4H, H_{arom}), 6.48 (s, 4H, H_{arom}), 6.31 (s, 2H, $CH_{methylene}$), 5.18 (s, 2H, N-CH_2), 4.11 (m, 12H, CH_2), 3.79 (t, J = 5 Hz, 8H, CH_2), 3.75 (t, J = 5.0 Hz, 4H, CH_2), 3.68 (m, 12H, CH_2), 3.61 (m, 24H, CH_2), 3.52 (m, 12H, CH_2), 3.351 (s, 6H, O-CH_3), 3.347 (s, 12H, O-CH_3) ppm; ^{13}C NMR (100 MHz, $CDCl_3$): δ = 174.2, 164.5, 162.7, 161.0, 153.1, 141.3, 138.4, 128.9. 128.2, 127.9, 125.0, 122.4, 112.3, 106.8, 87.7, 72.2, 71.8, 70.6, 70.5, 70.4, 70.3, 69.6, 68.9, 59.0, 49.9 ppm; HRMS (ESI, pos. mode, MeCN/$CHCl_3$): m/z: 1577.68018 $[M+H]^+$ (calcd for $C_{79}H_{109}N_4O_{25}S_2$ 1577.68168). Elemental analysis (%) calculated for $C_{79}H_{108}N_4O_{25}S_2$: C 60.14, H 6.9, N 3.55, S 4.06; found: C 60.14, H 7.04, N 3.76, S 3.78.

3. Optical studies in organic solvents

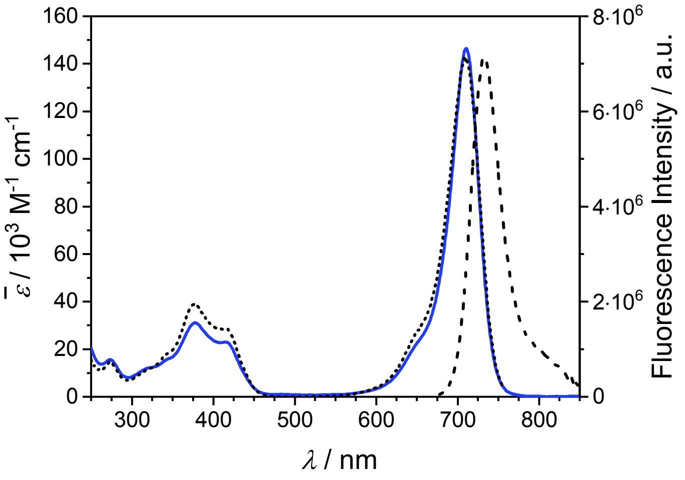

Figure S1 Absorption (filled), emission (dashed) and excitation (dotted) spectra of **CAS-C1** in chloroform solution.

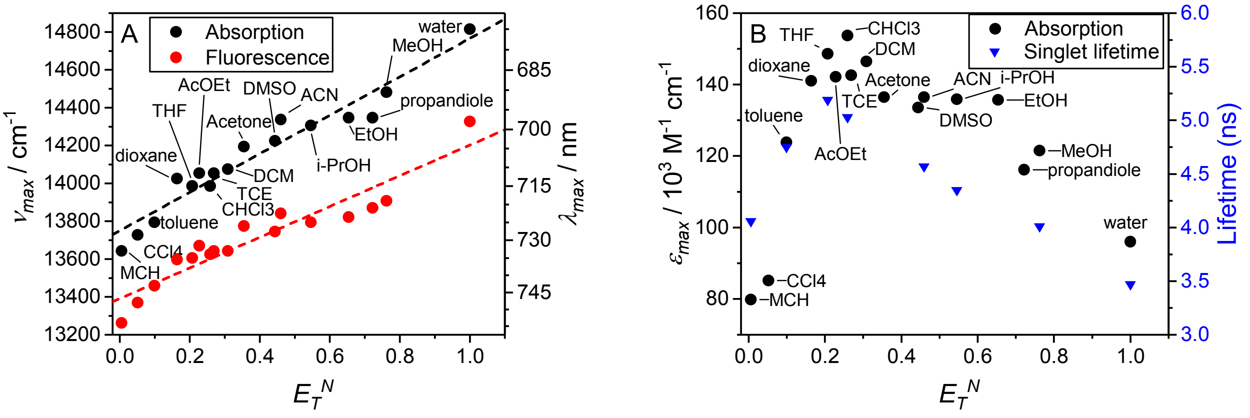

Figure S2 Change of the absorption (black) and emission (red) maxima for the optical spectra of **CAS-C1** depending on the solvent polarity. B) Change of the extinction coefficient (left axis, black) and fluorescence lifetime (right axis, blue) with the solvent polarity.

4. Optical studies in aqueous media

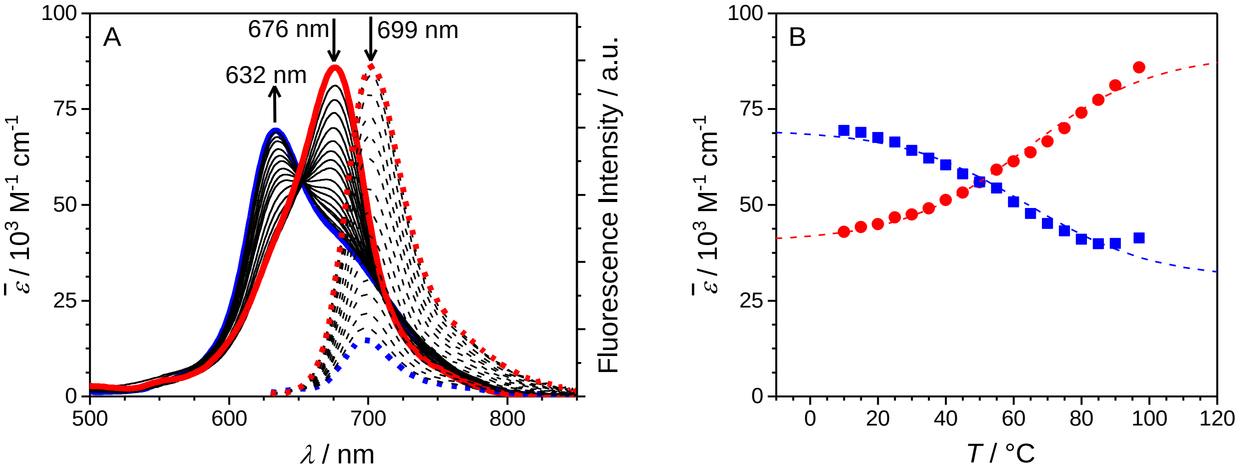

Figure S3 (A) UV/Vis (filled line) and fluorescence (dotted line) spectrum of an aqueous **CAS-C1** solution (5.8×10^{-7} M) upon decreasing the temperature from 95 (red) to 10 °C (blue). (B) Plot of the apparent extinction coefficient $\bar{\varepsilon}$ at 632 and 676 nm against the temperatures. Dotted lines correspond to a fitting with the isodesmic model (T_m = 64 °C, ΔH^{pol} = -55.9 kJ mol^{-1}, R^2 = 0.985).

The apparent extinction coefficient was plotted against the temperature and fitted well with a temperature-dependent isodesmic aggregation model (Eq. S3),[22] with an estimated T_m = 64 °C and assuming an enthalpy of each step of polymerization of ΔH^{pol} = - 55.9 kJ mol^{-1}.

$$\alpha_{agg}^{\lambda} = \frac{\varepsilon_{(T)}^{\lambda} - \varepsilon_{(low)}^{\lambda}}{\varepsilon_{(high)}^{\lambda} - \varepsilon_{(low)}^{\lambda}} = \frac{1}{1 + e^{-0.908\, \Delta H^{pol} \frac{T - T_m}{R\,(T_m + 273.15)^2}}} \qquad \text{(Eq. S3)}$$

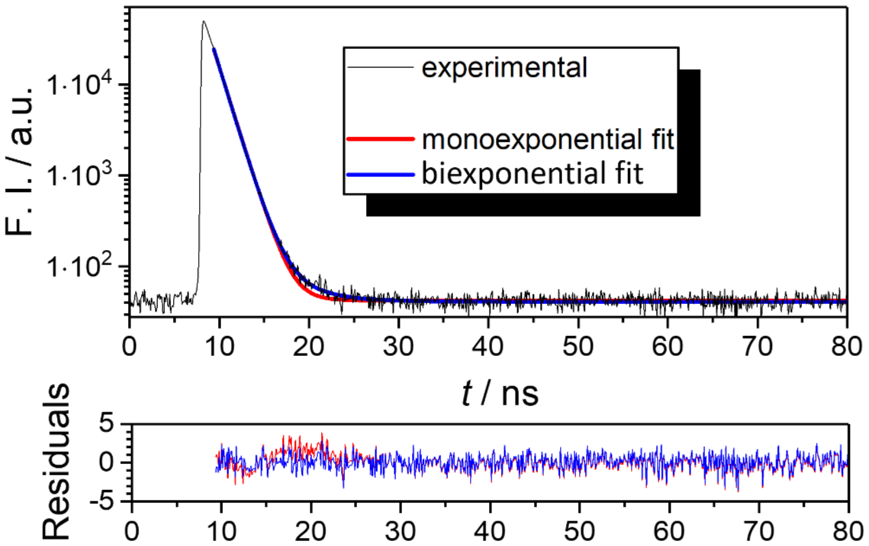

Figure S4 Time-correlated single photon counting decay (excitation at 670 nm) for **CAS-C1** in water at 80 °C. In red the fitting with a monoexponential function (Eq. S1); the fitting with a biexponential function (Eq. S2, τ_1 = 4.76 ns, τ_2 = 1.35 ns). The fitting with the biexponential function better describes the decay in the range 15 – 25 s (R^2 = 0.9998) compared with the monoexponential function (R^2 = 0.9972).

5. G-quadruplex studies

Kinetics

The binding of **CAS-C1** with various G4s revealed no significant kinetics.

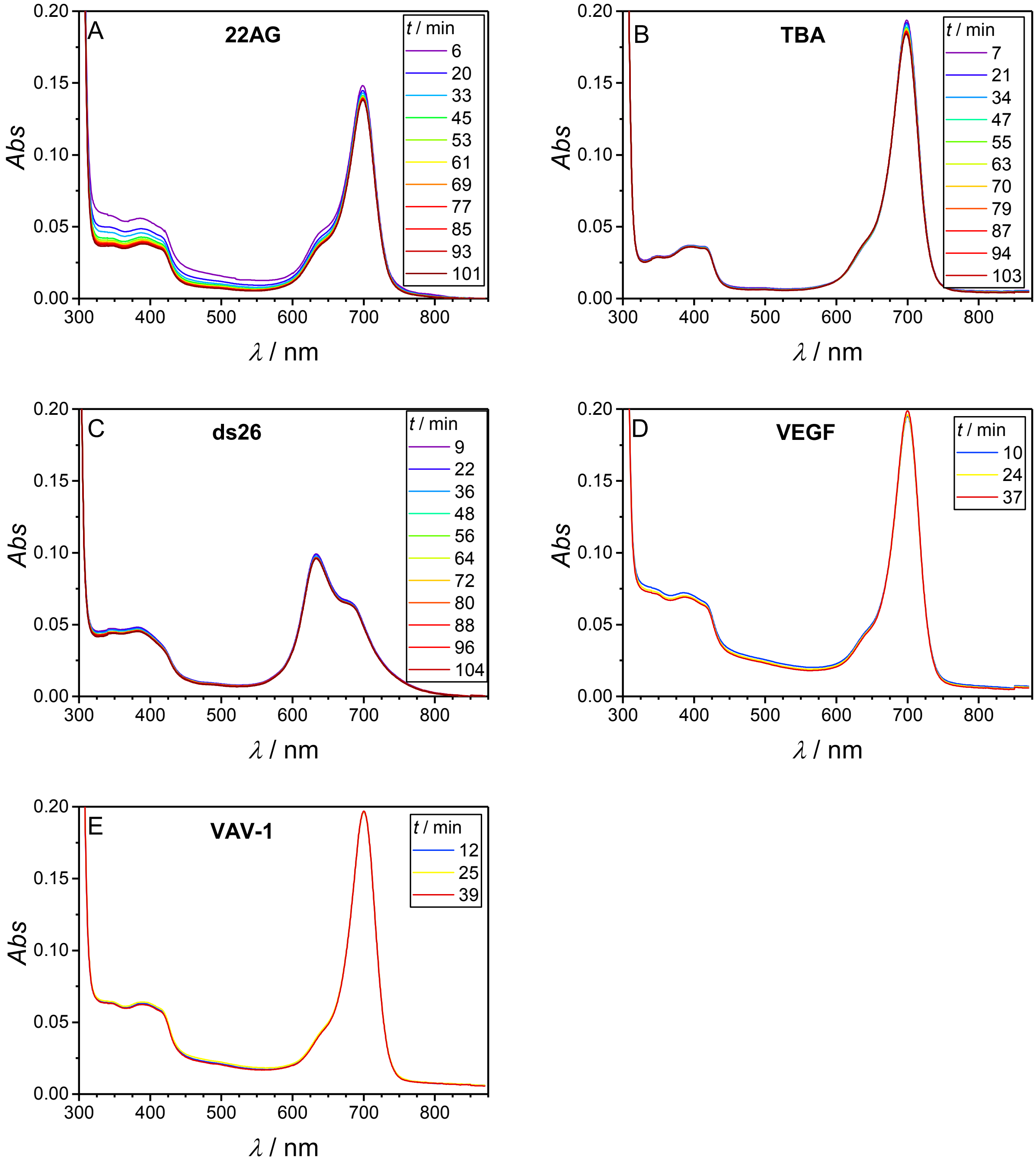

Figure S5 Kinetics of interaction between **CAS-C1** and: A) 22AG; B) TBA; C) ds26; D) VEGF and E) VAV-1. (TRIS buffer *c* = 10 mM, pH = 7.2, KCl *c* = 100 mM, 25 °C). After addition of G4, the sample was measured at different time intervals as indicted in the insets.

G-quadruplex titrations

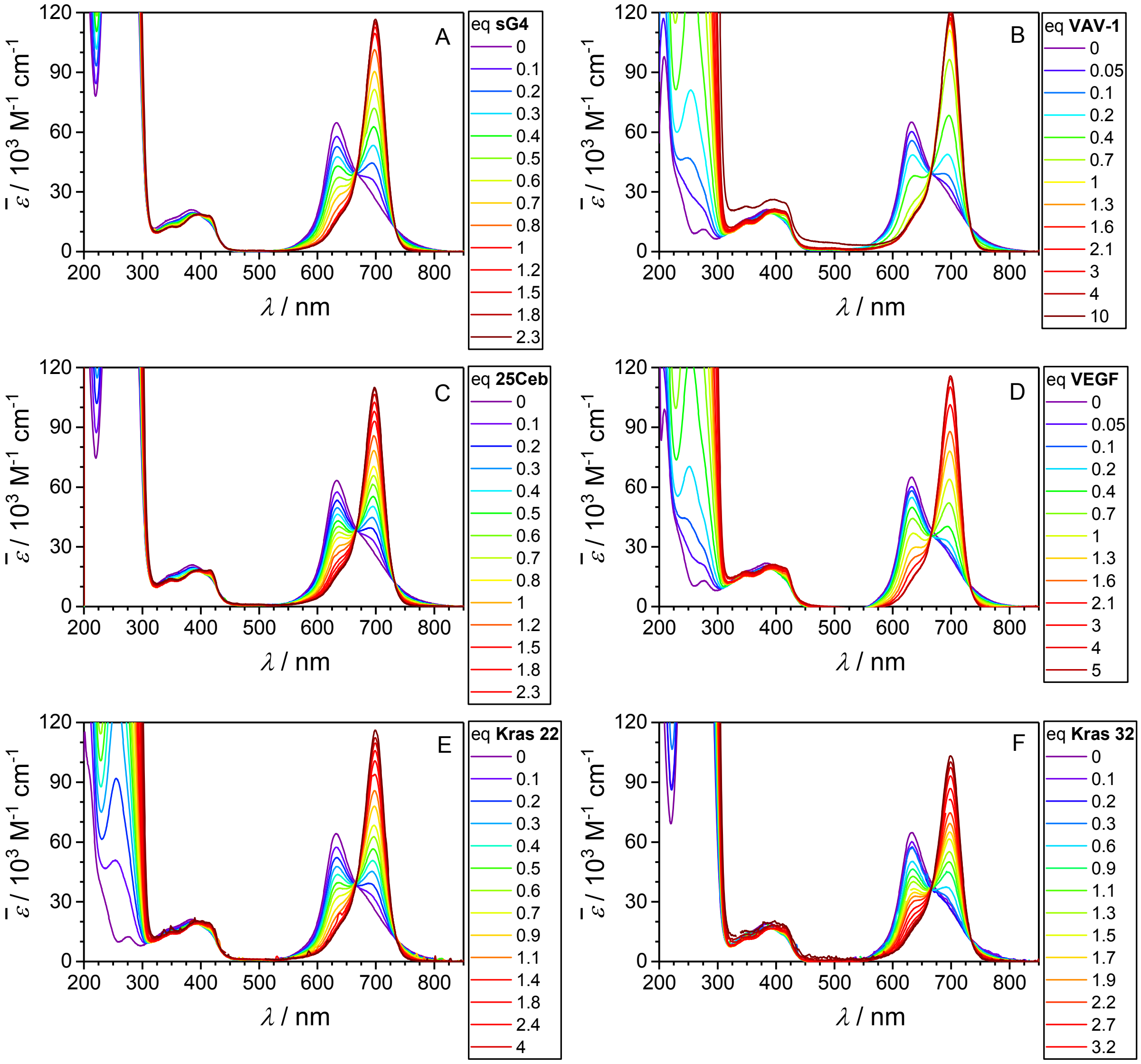

Figure S6 UV/Vis spectra of **CAS-C1** in buffered water solution (1.66×10^{-6} M, TRIS buffer c = 10 mM, pH = 7.2, KCl c = 100 mM) upon addition of various G4s (500 µM, buffered solution) at 25 °C: A) sG4, B) VAV-1, C) 25Ceb, D) VEGF, E) Kras 22 and F) Kras 32.

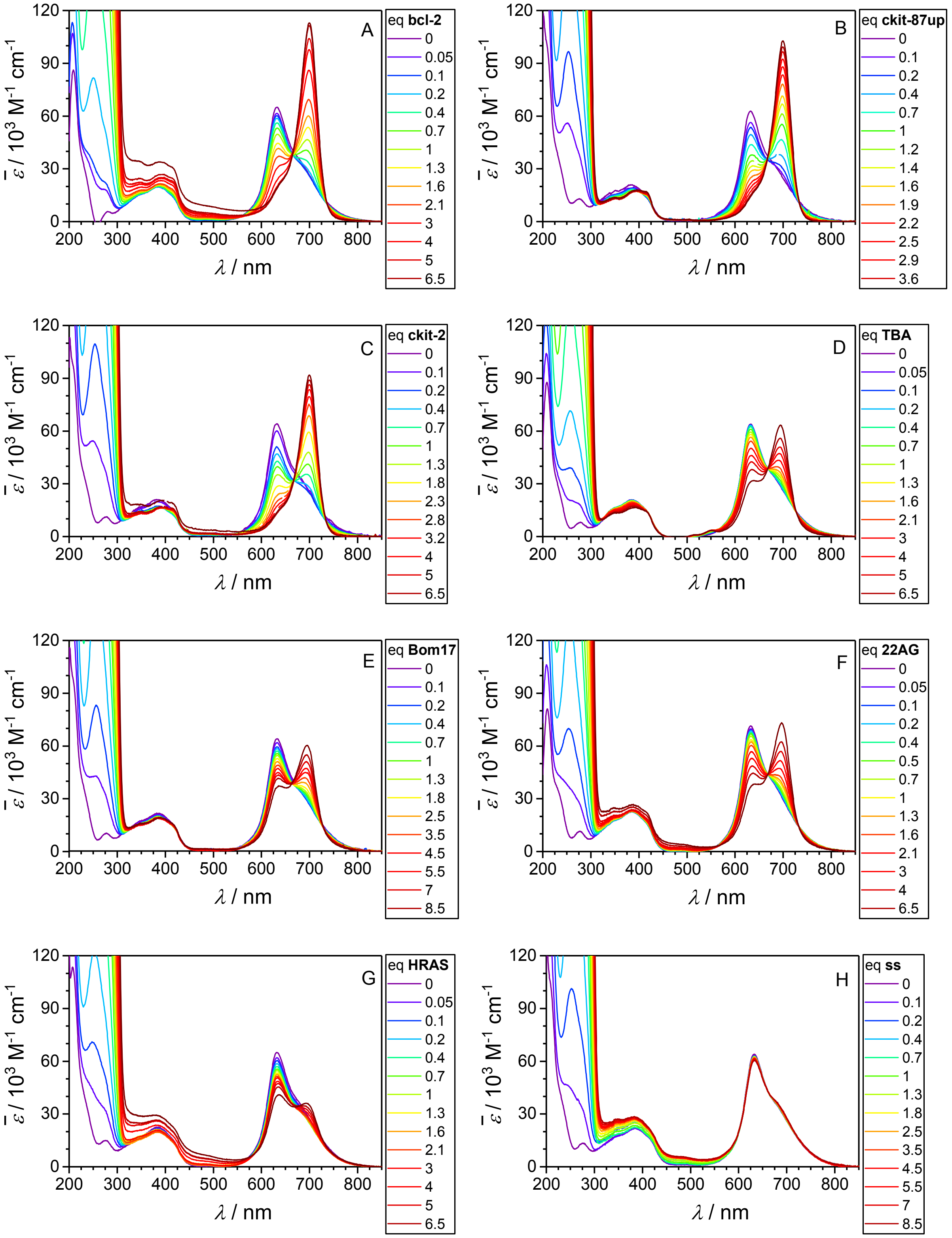

Figure S7 UV/Vis spectra of **CAS-C1** in buffered water solution (1.66×10^{-6} M, TRIS buffer c = 10 mM, pH = 7.2, KCl c = 100 mM) upon addition of various G4s (500 µM, buffered solution) at 25 °C: A) bcl-2, B) ckit-87up, C) ckit-2, D) TBA, E) Bom17, F) 22AG, G) HRAS and H) ss.

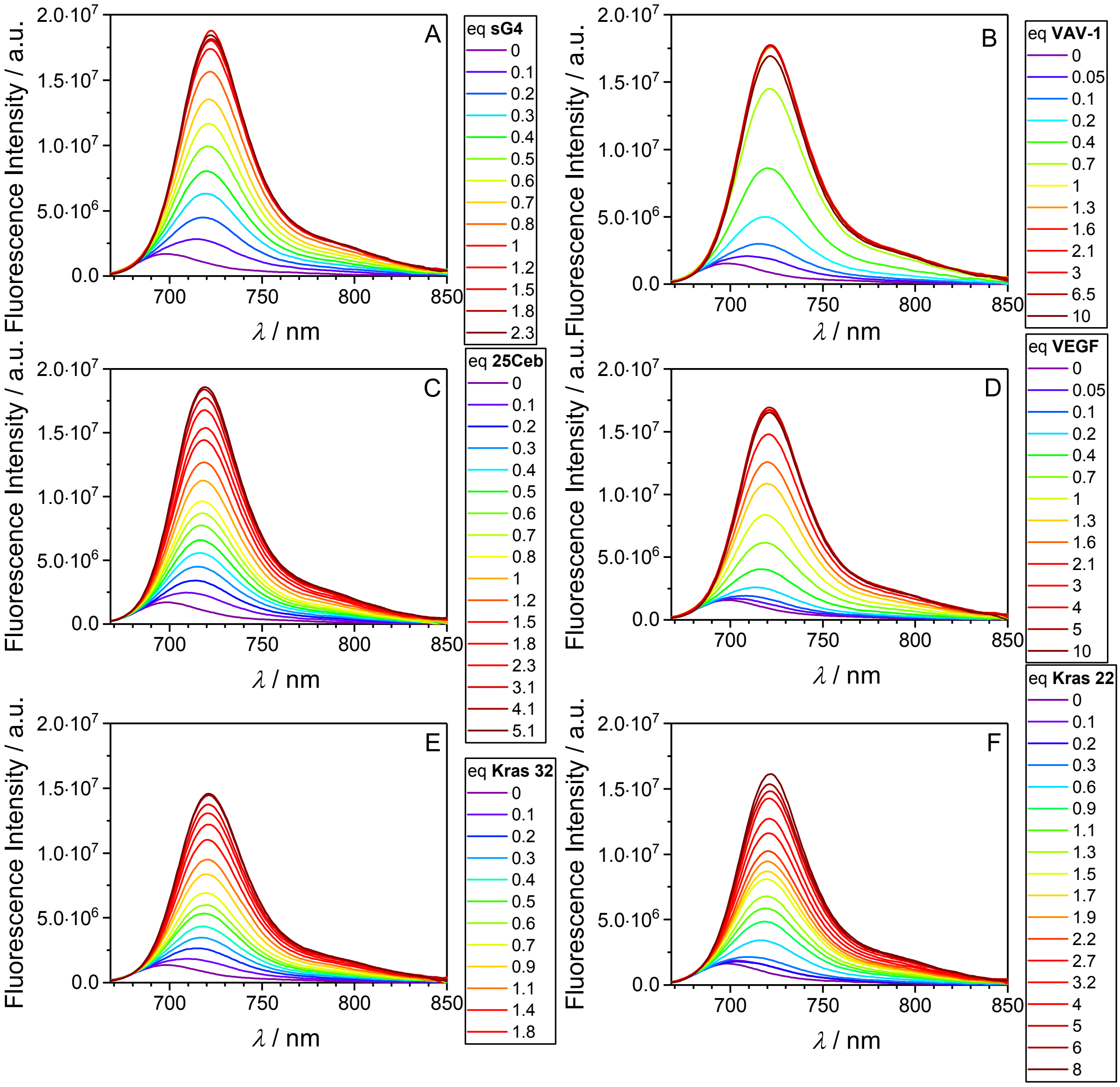

Figure S8 Fluorescence spectra (excitation at 660 nm) of **CAS-C1** in buffered water solution (1.66×10^{-6} M, TRIS buffer c = 10 mM, pH = 7.2, KCl c = 100 mM) upon addition of various G4s (500 µM, buffered solution) at 25 °C: A) sG4, B) VAV-1, C) 25Ceb, D) VEGF, E) Kras 22 and F) Kras 32.

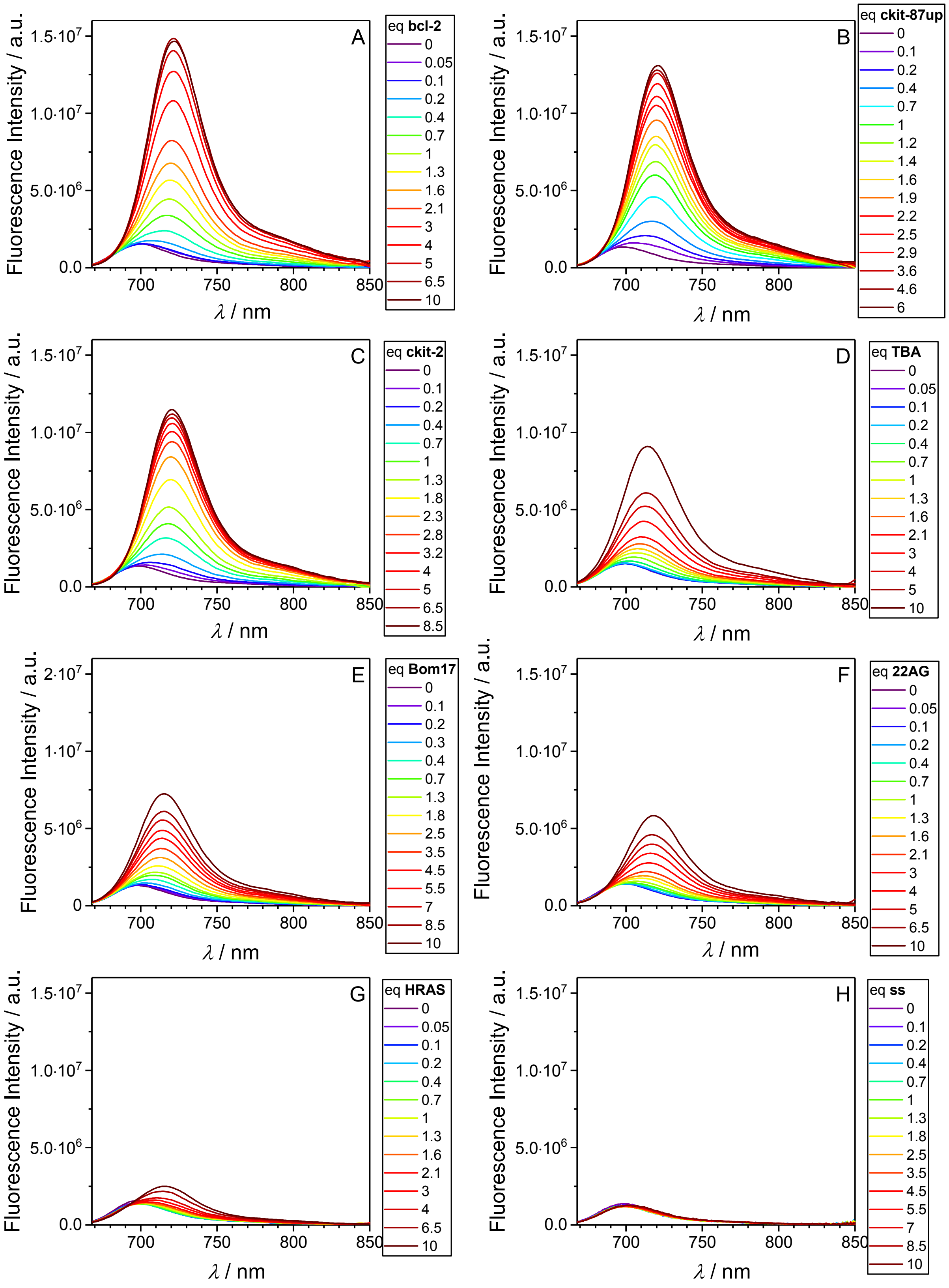

Figure S9 Fluorescence spectra (excitation at 660 nm) of **CAS-C1** in buffered water solution (1.66 × 10^{-6} M, TRIS buffer c = 10 mM, pH = 7.2, KCl c = 100 mM) upon addition of various G4s (500 µM, buffered solution) at 25 °C: A) bcl-2, B) ckit-87up, C) ckit-2, D) TBA, E) Bom17, F) 22AG, G) HRAS and H) ss.

Photophysical characterization

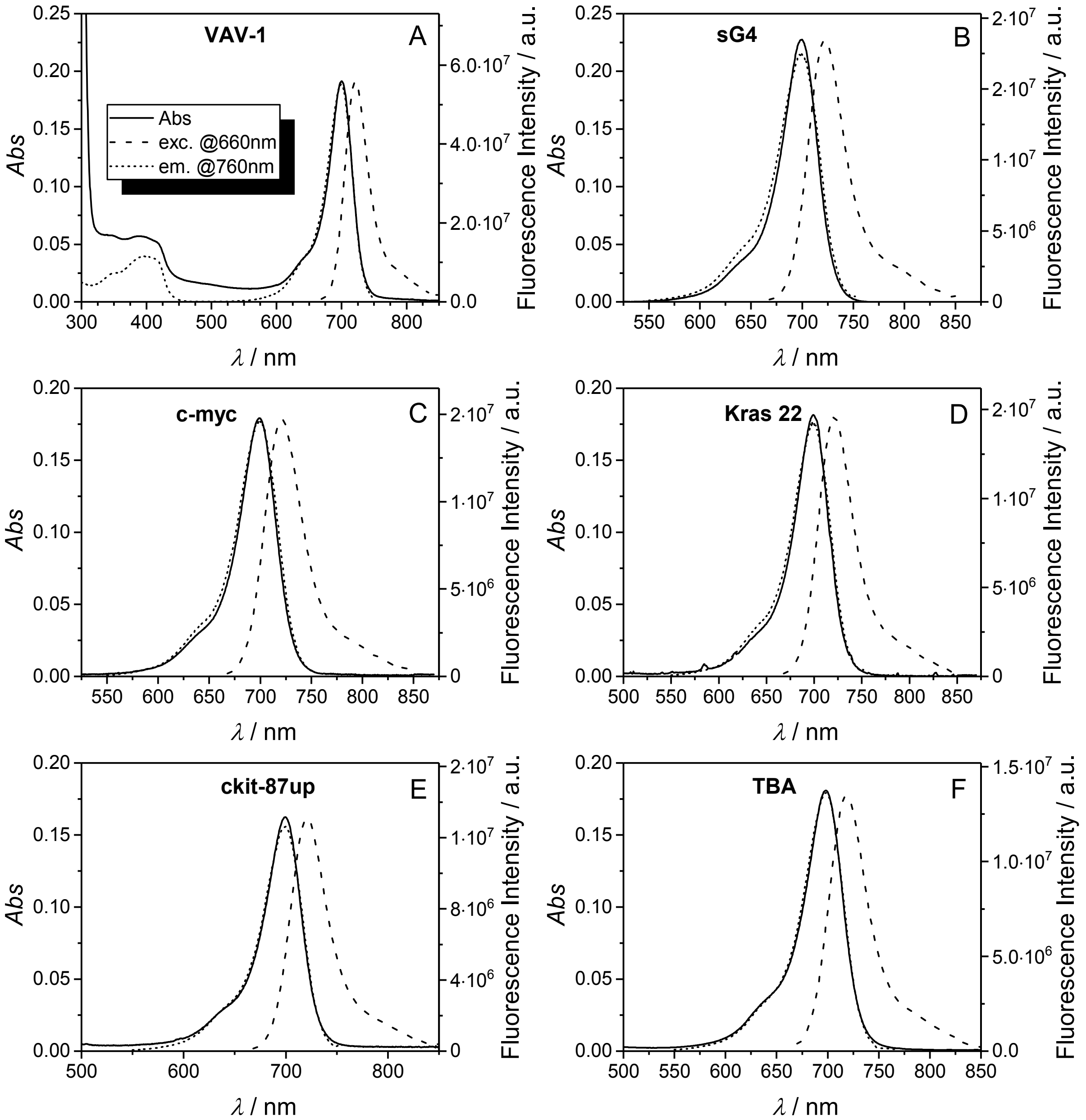

Figure S10 Absorption (filled line), emission (excitation at 660 nm, dashed) and excitation spectra (emission at 760 nm, dotted) of **CAS-C1** complex with: A) 14 eq VAV-1, B) 2.9 eq sG4, C) 5.1 eq c-myc, D) 10.8 eq Kras 22, E) 9.3 eq ckit-87up and F) 25 eq TBA.

Fluorescence lifetimes

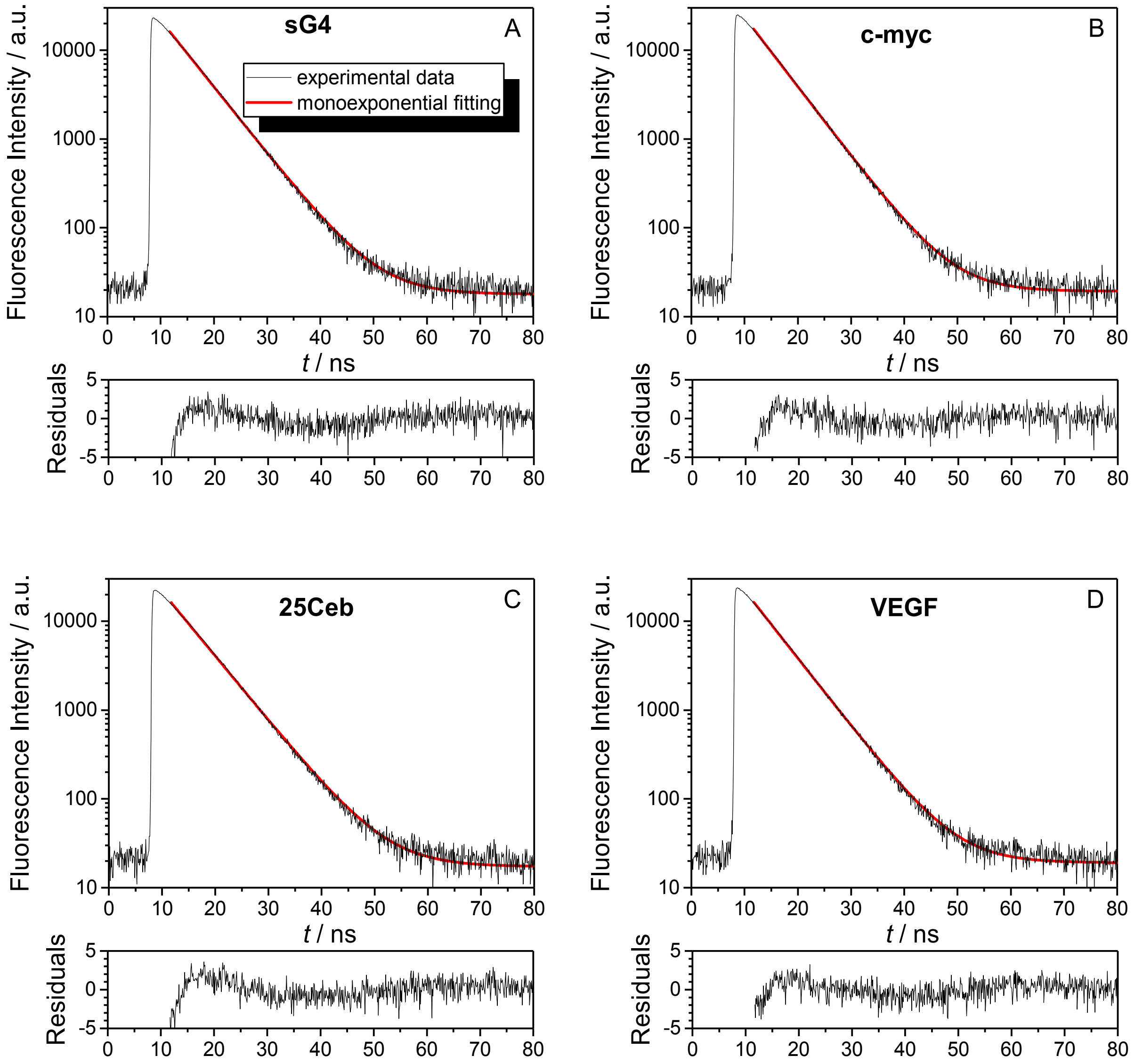

Figure S11 Time-correlated single photon counting decay (excitation at 670 nm) for **CAS-C1** with addition of A) 2.9 eq sG4, B) 5.1 eq c-myc, C) 6.1 eq 25Ceb, D) 14.5 eq VEGF (TRIS buffer *c* = 10 mM, *pH* = 7.2, KCl *c* = 100 mM, 25 °C). In red is shown the monoexponential fitting. The lifetime is shown in Table 1 (main text).

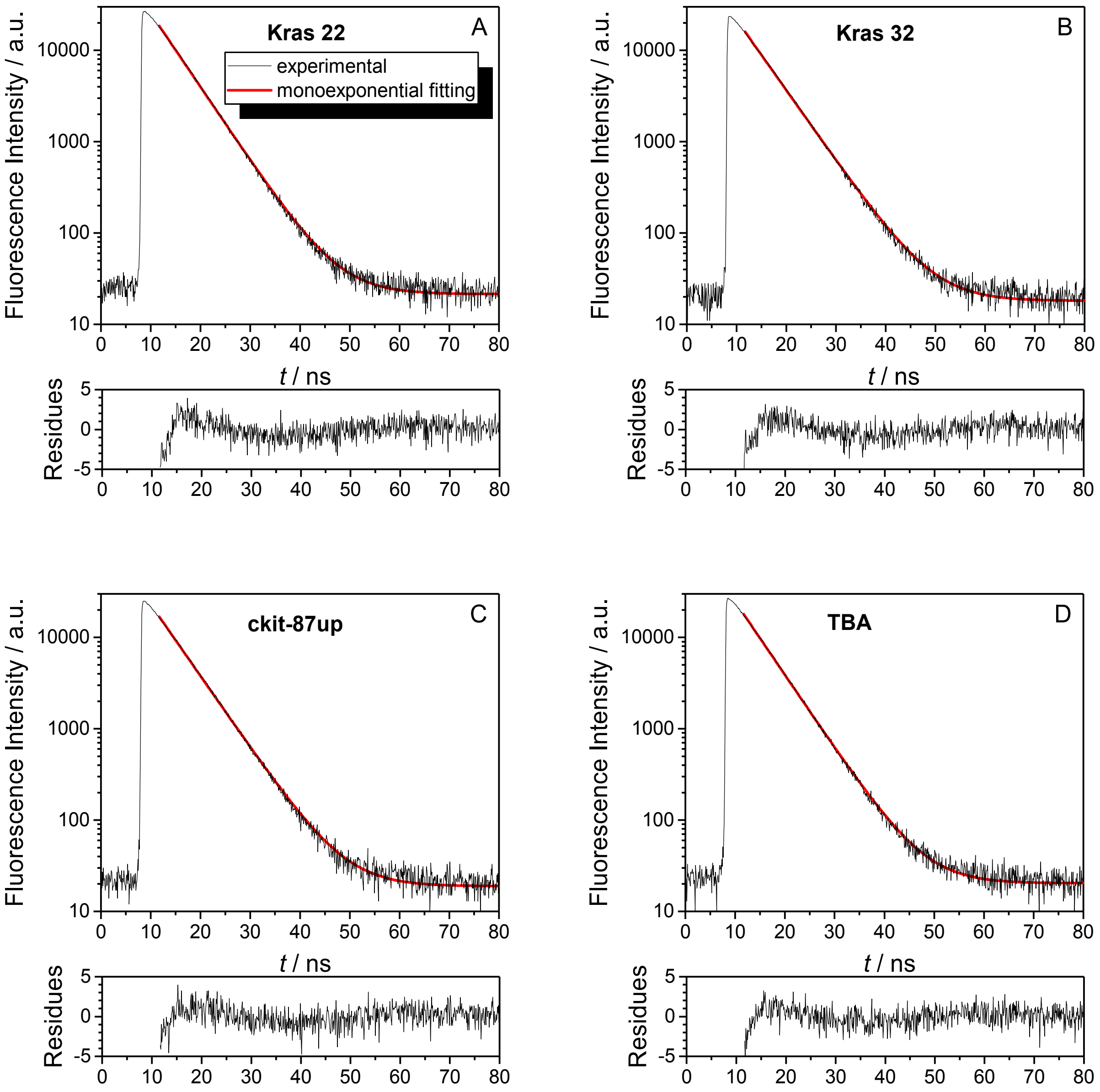

Figure S12 Time-correlated single photon counting decay (excitation at 670 nm) for **CAS-C1** with addition of A) 10.8 eq Kras 22, B) 9.2 eq Kras 32, C) 9.4 eq ckit-87up, D) 25 eq TBA (TRIS buffer *c* = 10 mM, *pH* = 7.2, KCl *c* = 100 mM, 25 °C). In red is shown the monoexponential fitting. The lifetime is shown in Table 1 (main text).

Job´s plot and stoichiometry

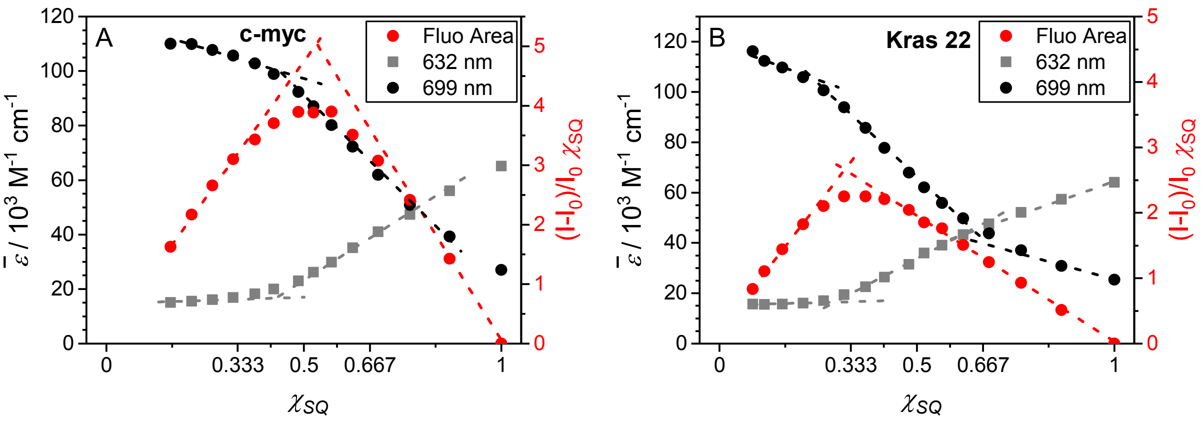

Figure S13 Job´s plot for the titration of **CAS-C1** with A) c-myc and B) Kras 22 monitored in the absorption at the maximum wavelength of the aggregate (632 nm, grey) and the complex (699 nm, black) and the fluorescence total area (red).

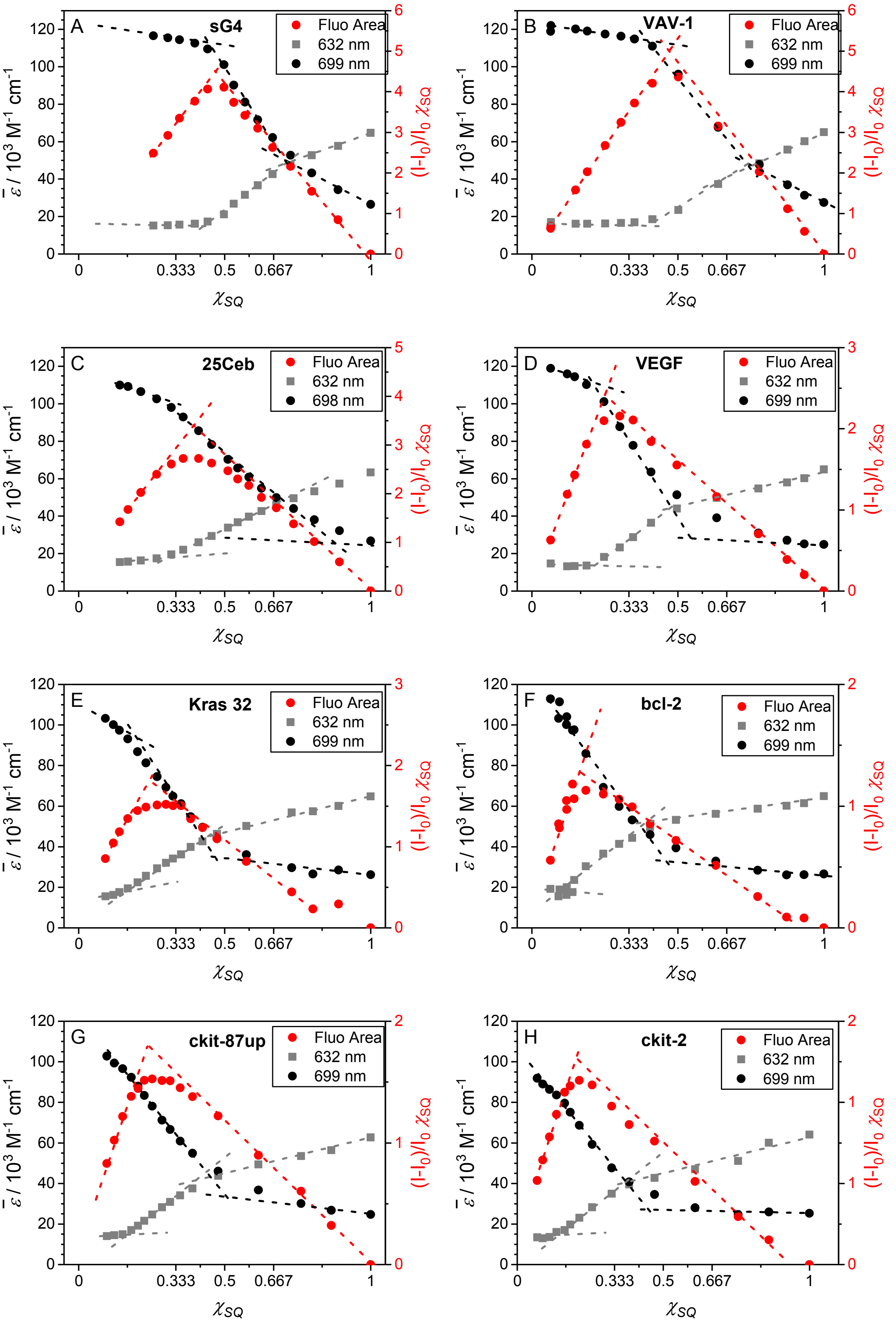

Figure S14 Job´s plot for the titration of **CAS-C1** with various G4s (A-H) monitored in the absorption at the maximum wavelength of the aggregate (632 nm, grey) and the complex (699 nm, black) and the fluorescence total area (red).

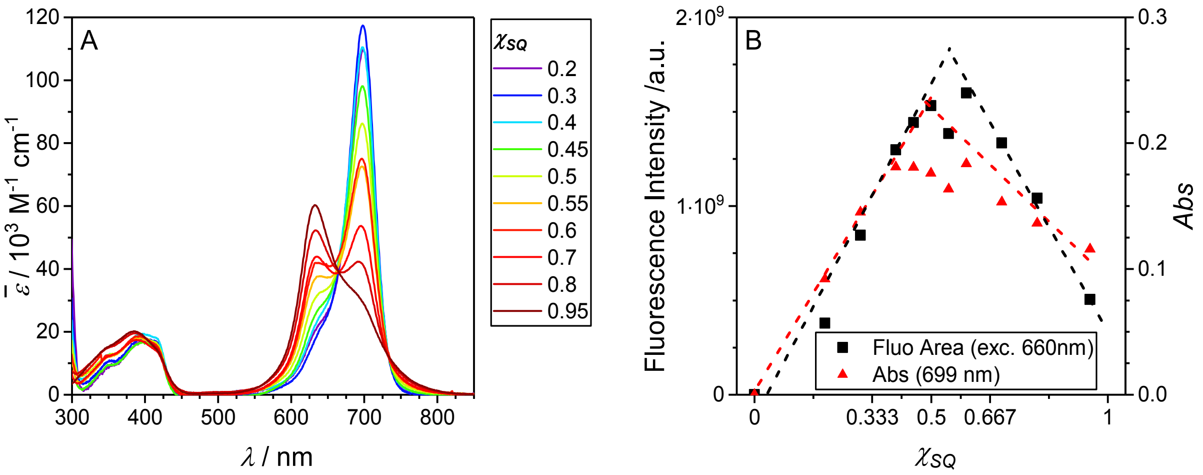

Figure S15 Job´s plot by independent experiments obtained by mixing **CAS-C1** and VAV-1 at different ratios with a total concentration ([**CAS-C1**]+[VAV-1] = 4.0 × 10^{-6} M): A) absorption spectra and B) Job´s plot obtained by monitoring the absorption at 699 nm and the fluorescence total area (excitation at 660 nm). (TRIS buffer *c* = 10 mM, pH = 7.2, KCl *c* = 100 mM, 25 °C)

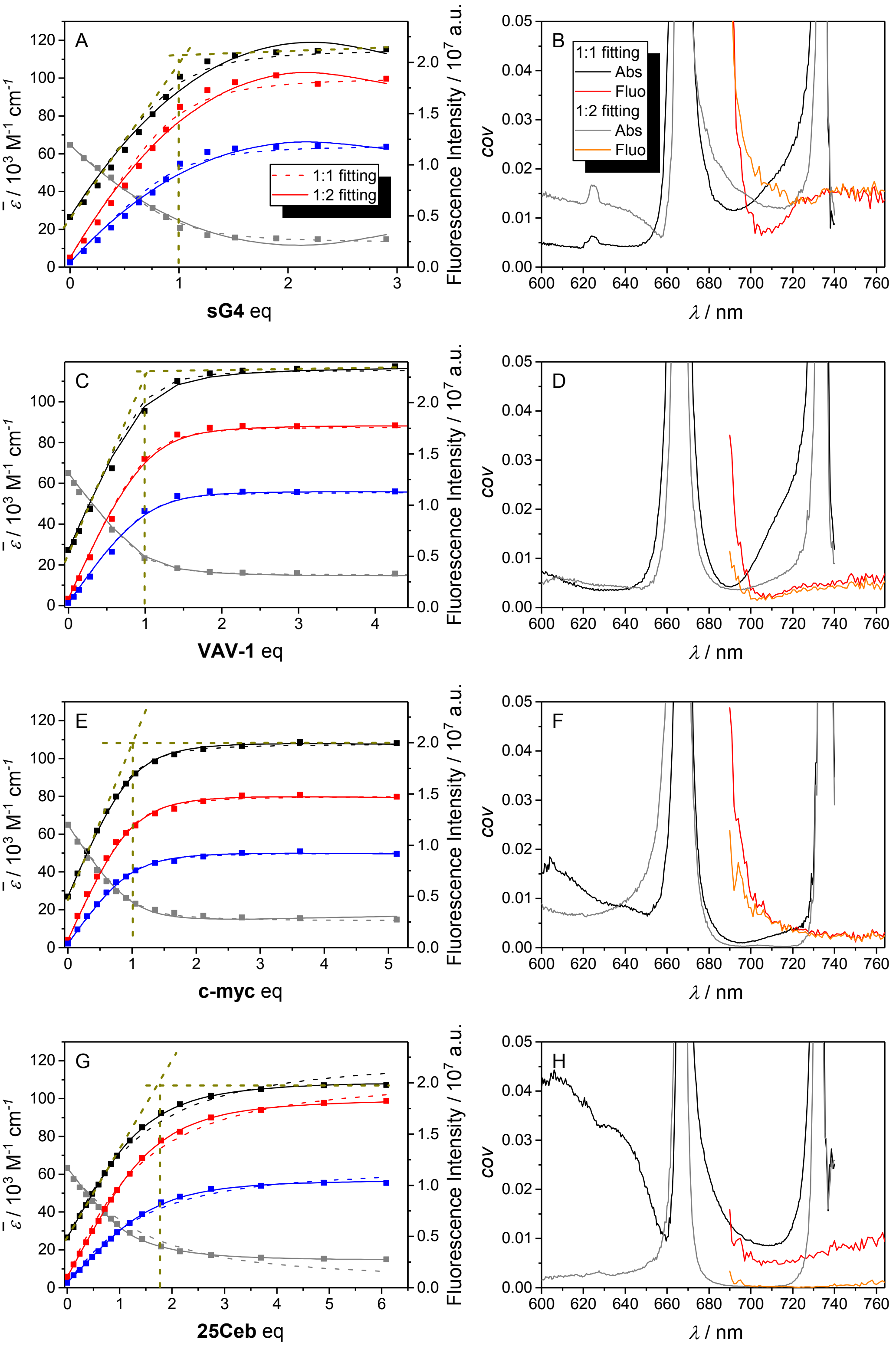

Figure S16 Global fitting of **CAS-C1** titration with A) sG4, C) VAV-1, E) c-myc, G) 25Ceb monitored in the absorption spectra at 632 nm (aggregate maximum wavelength, grey), 699 nm (complex maximum, black) and in the fluorescence spectra at the maximum emission wavelength (red) and at 740 nm (blue). The fitting is shown for 1:1 (dotted) and 1:2 (filled) binding model. The covariance is shown on the right (B, D, F, H for the respective G4s) for the 1:1 fitting of the absorption (black) and fluorescence (red) data, and the 1:2 fitting (grey for the absorption and orange for the fluorescence).

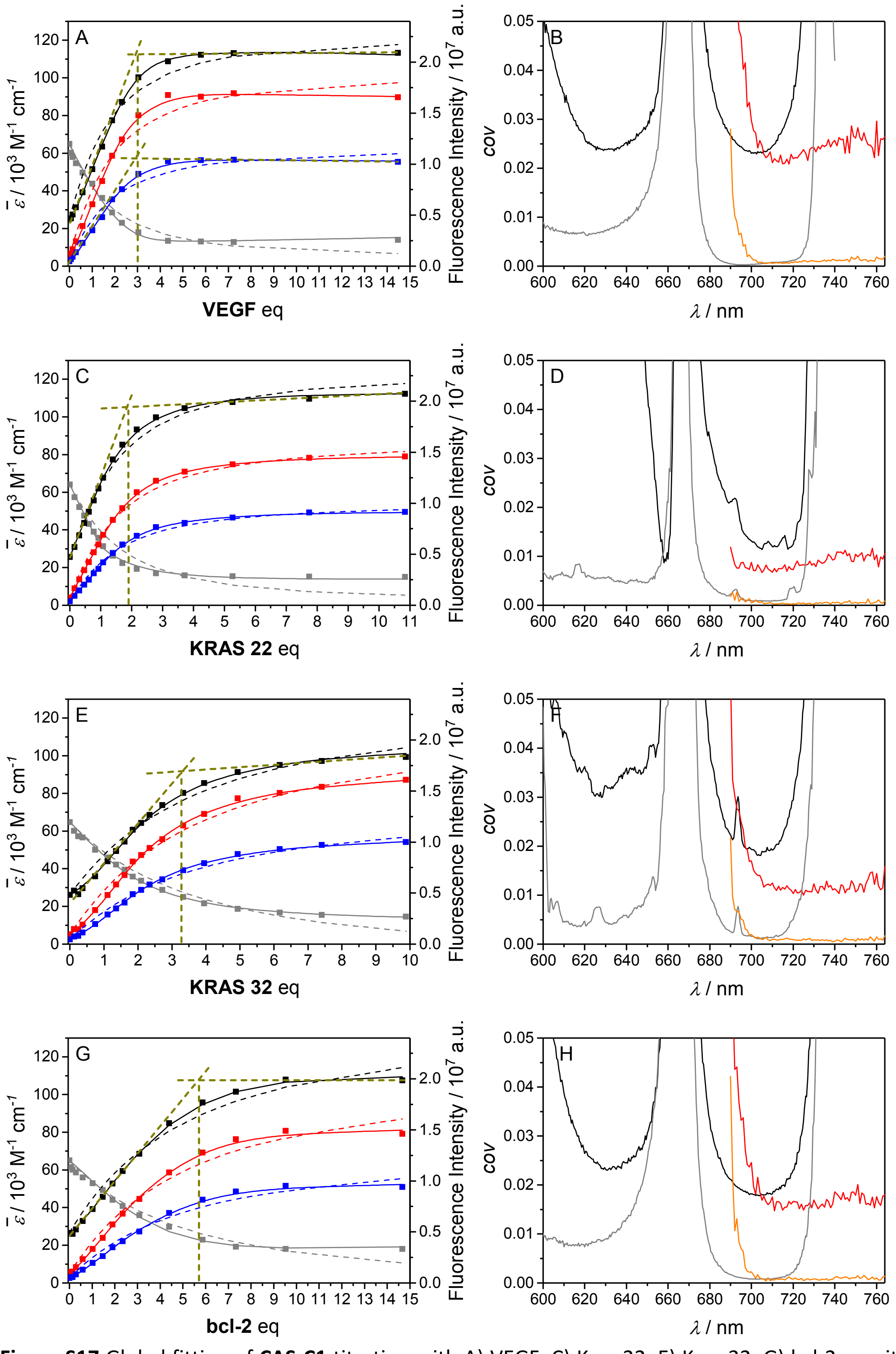

Figure S17 Global fitting of **CAS-C1** titration with A) VEGF, C) Kras 22, E) Kras 32, G) bcl-2 monitored in the absorption spectra at 632 nm (aggregate maximum wavelength, grey), 699 nm (complex maximum, black) and in the fluorescence spectra at the maximum emission wavelength (red) and at 740 nm (blue). The fitting is shown for 1:1 (dotted) and 1:2 (filled) binding model. The covariance is shown on the right (B, D, F, H for the respective G4s) for the 1:1 fitting of the absorption (black) and fluorescence (red) data, and the 1:2 fitting (grey for the absorption and orange for the fluorescence).

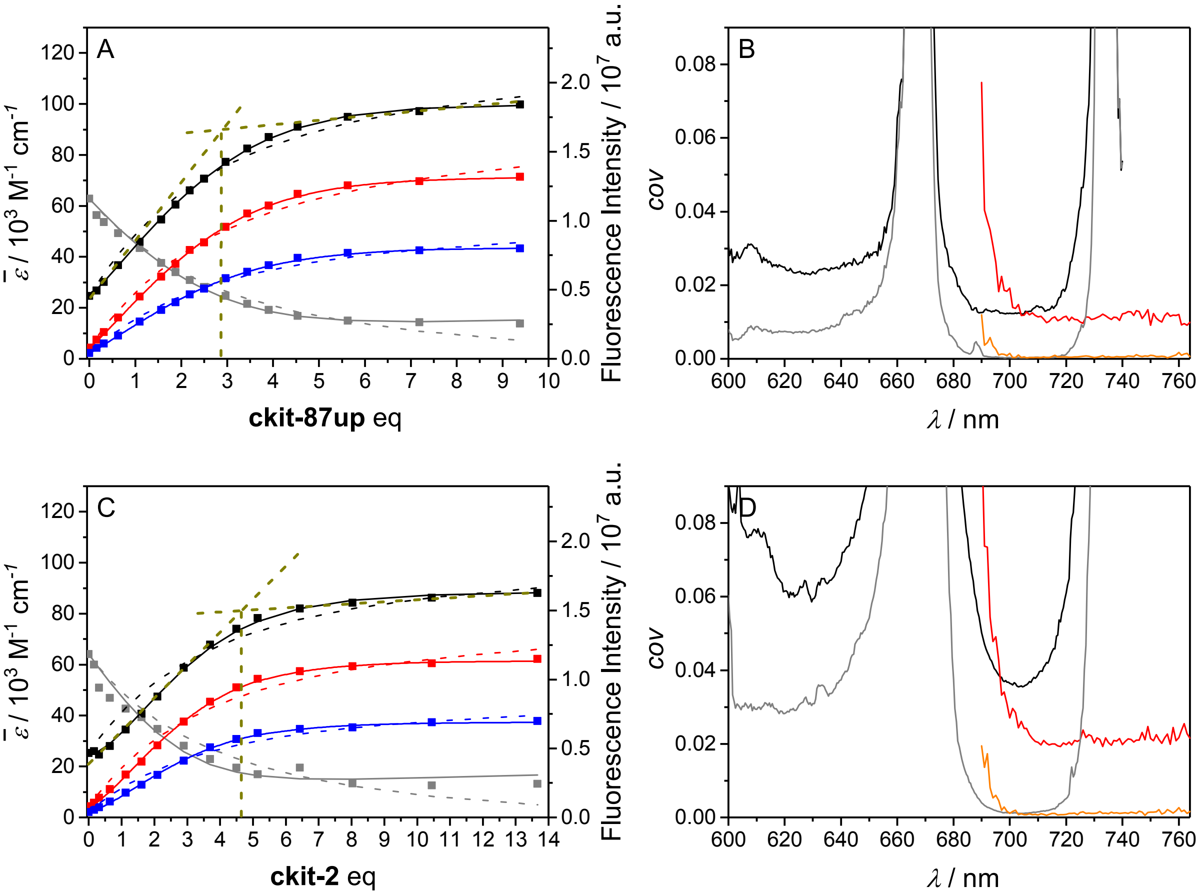

Figure S18 Global fitting of **CAS-C1** titration with A) ckit-87up, C) ckit-2 monitored in the absorption spectra at 632 nm (aggregate maximum wavelength, grey), 699 nm (complex maximum, black) and in the fluorescence spectra at the maximum emission wavelength (red) and at 740 nm (blue). The fitting is shown for 1:1 (dotted) and 1:2 (filled) binding model. The covariance is shown on the right (B, D for the respective G4s) for the 1:1 fitting of the absorption (black) and fluorescence (red) data, and the 1:2 fitting (grey for the absorption and orange for the fluorescence).

Competition titrations

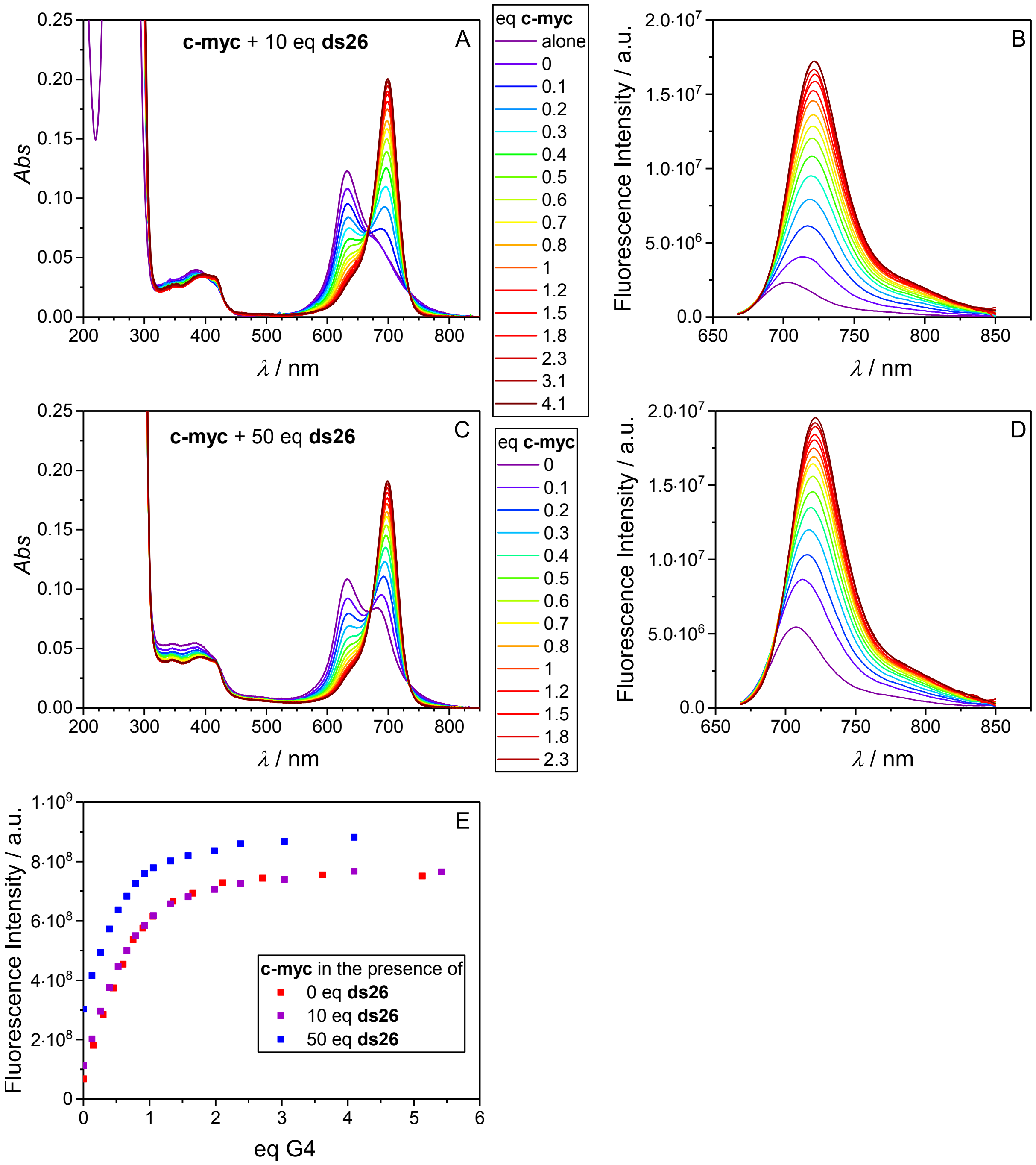

Figure S19 Spectrophotometric (A and C) and fluorimetric (B and D) titration of **CAS-C1** in buffered water solution (1.66×10^{-6} M, TRIS buffer c = 10 mM, pH = 7.2, KCl c = 100 mM) with c-myc (500 µM, buffered solution) at 25 °C in the presence of ds26 DNA: A-B) 10 eq, C-D) 50 eq. E) Comparison of the fluorescence total area (excitation at 660 nm) of the two experiments and the one in the absence of ds26 DNA. (b = 1.00 cm).

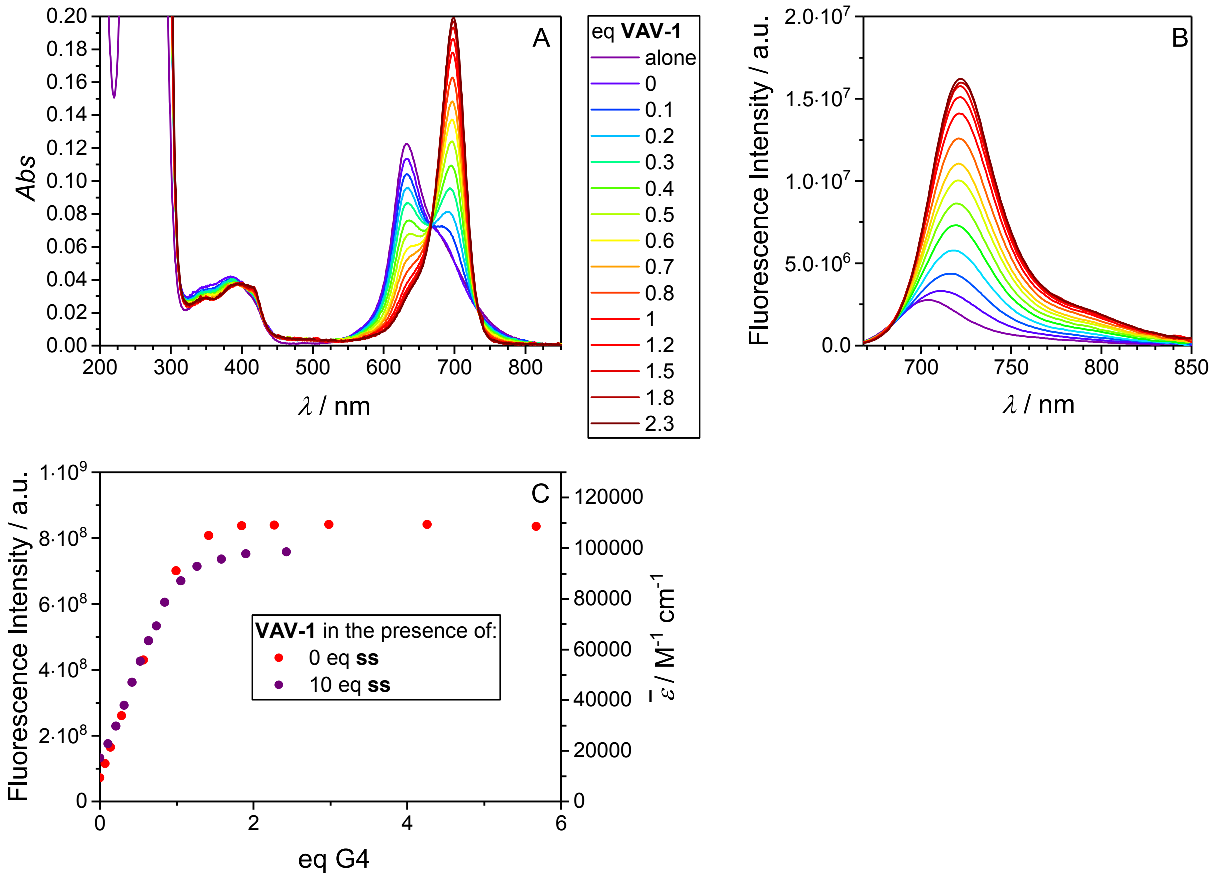

Figure S20 Spectrophotometric (A) and fluorimetric (B) titration of **CAS-C1** in buffered water solution (1.66×10^{-6} M, TRIS buffer c = 10 mM, pH = 7.2, KCl c = 100 mM) with VAV-1 (500 µM, buffered solution) at 25 °C in the presence of 10 eq ds26 DNA. C) Comparison of the fluorescence total area (excitation at 660 nm) of the two experiments and the one in the absence of ds26 DNA. (b = 1.00 cm).

Speciation analysis

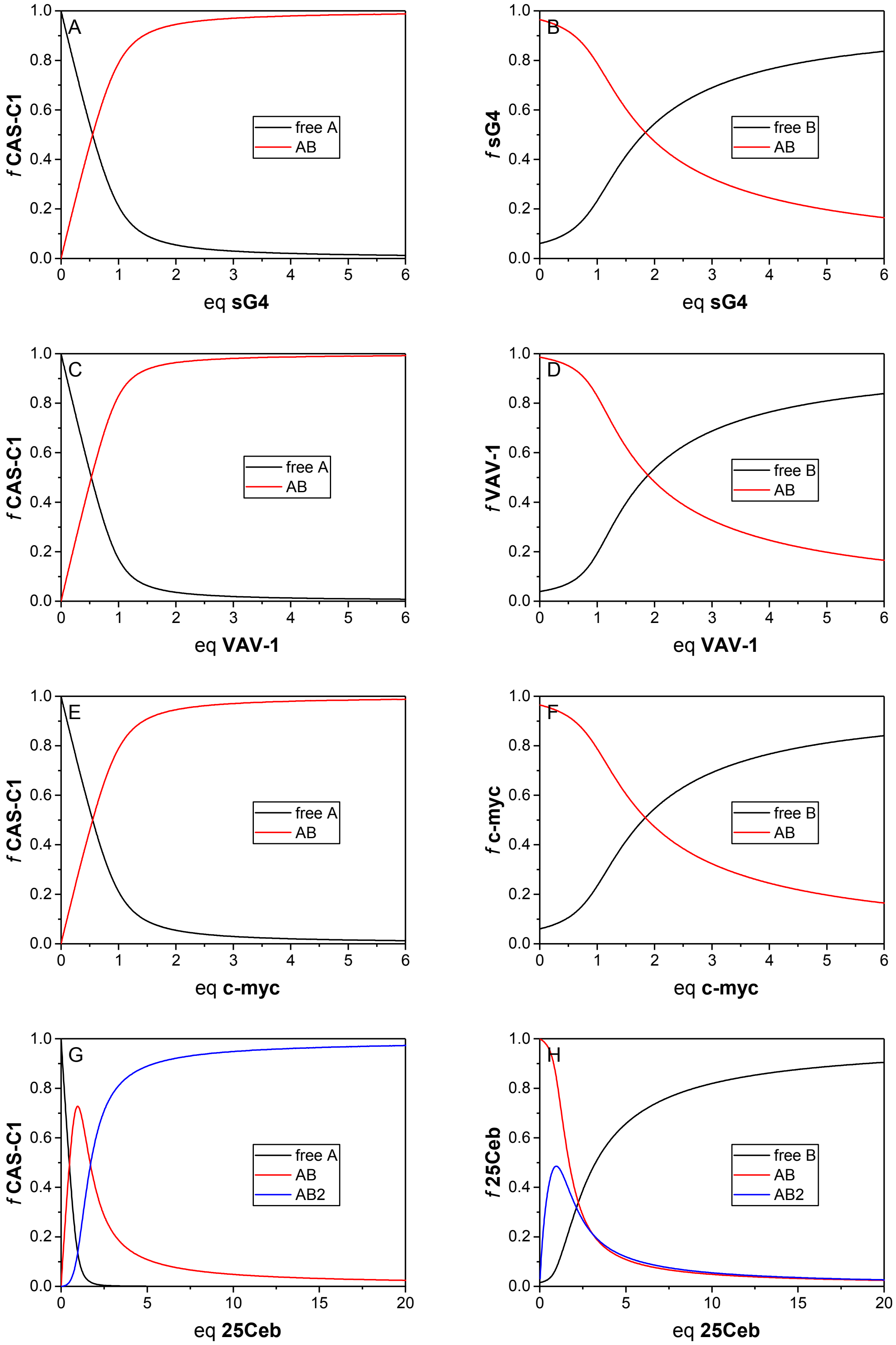

Figure S21 Speciation analysis for the titrations of **CAS-C1** with G4s utilizing the values of K_{11} (and K_{12}, when suitable) obtained by fitting as described in the references.[14,15]

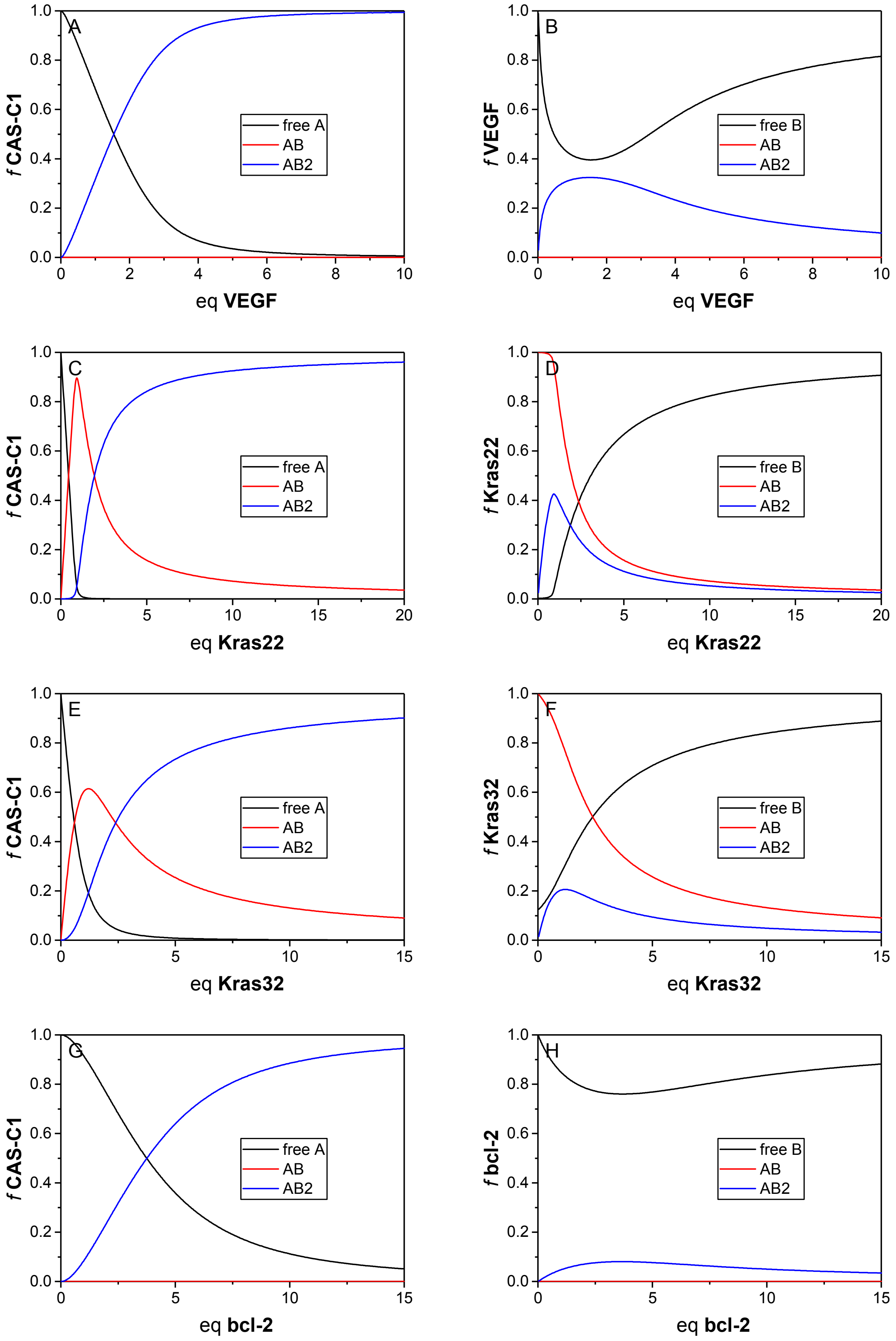

Figure S22 Speciation analysis for the titrations of **CAS-C1** with G4s utilizing the values of K_{11} (and K_{12}, when suitable) obtained by fitting as described in the references.[14,15]

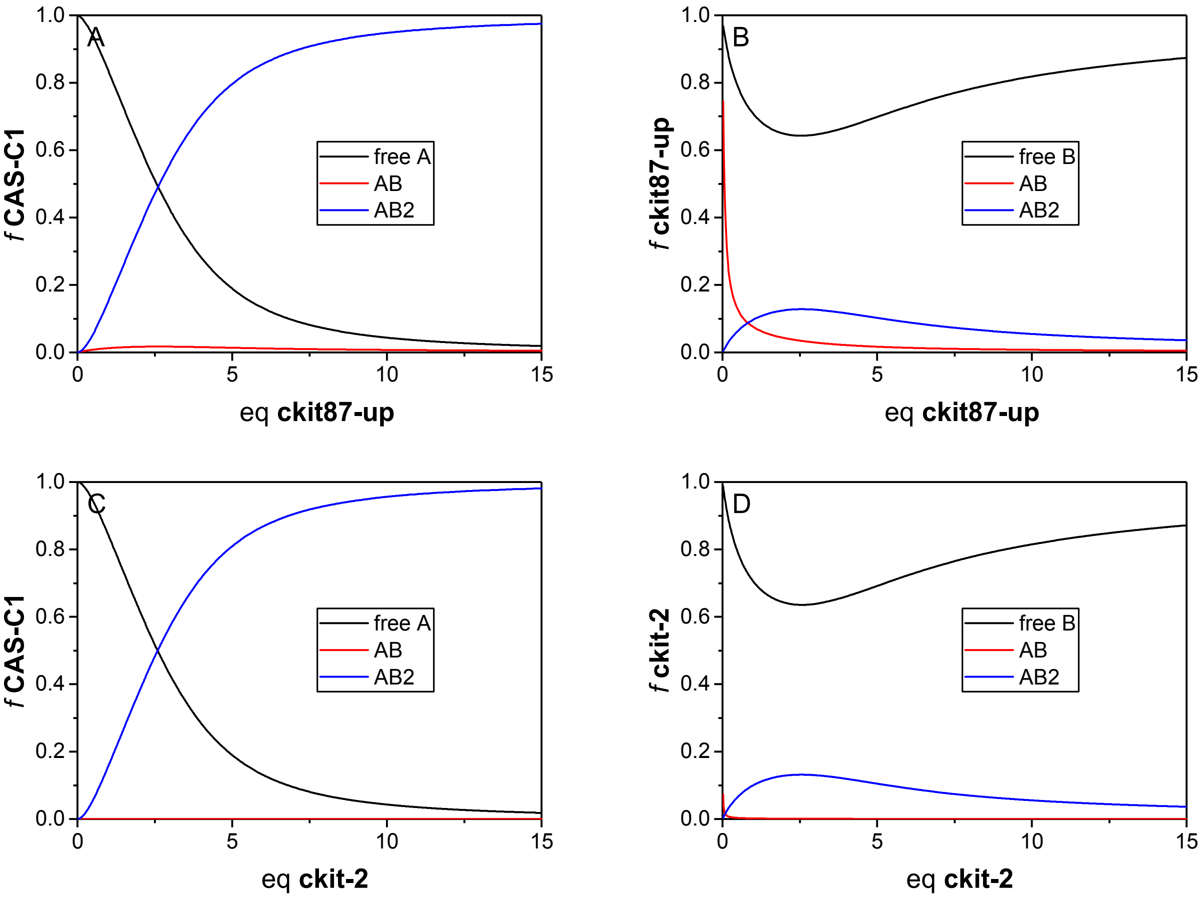

Figure S23 Speciation analysis for the titrations of **CAS-C1** with G4s utilizing the values of K_{11} (and K_{12}, when suitable) obtained by fitting as described in the references.[14,15]

Two-photon absorption

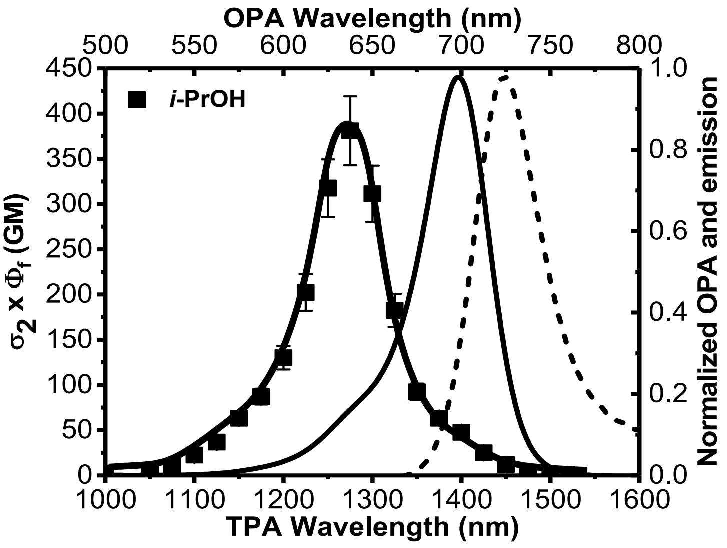

Figure S24 Molecular brightness ($\sigma_2 \times \Phi_F$) plot of **CAS-C1** in *i*-PrOH.

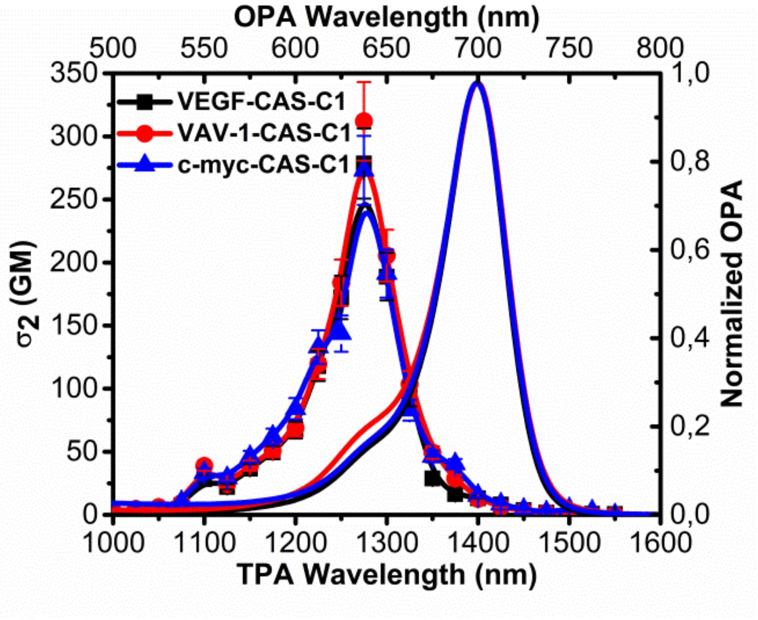

Figure S25 Two-photon absorption cross-section (σ_2) of **CAS-C1** in the presence of VEGF, VAV-1 and c-myc (10 mM Tris, 100 mM K^+, pH 7.2) at molar ratio (*r*) = 7. The molecular brightness is shown in Fig. 4.

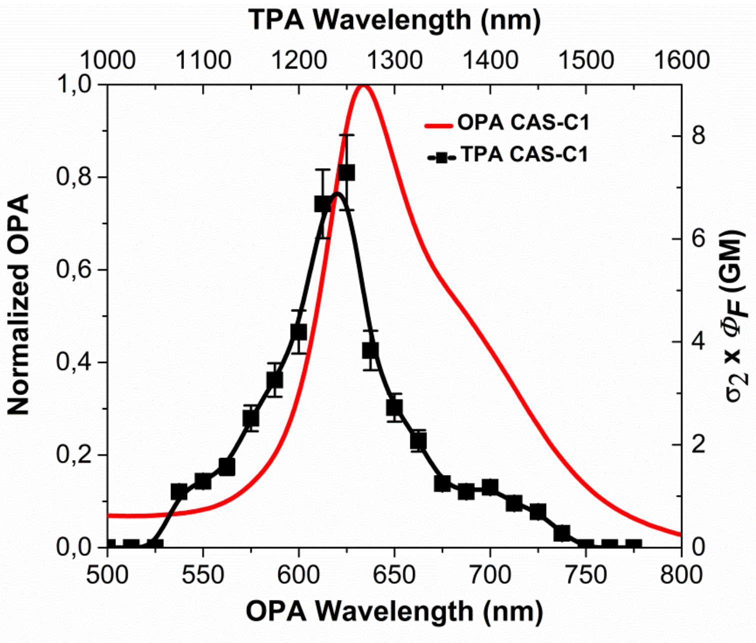

Figure S26 Molecular brightness ($\sigma_2 \times \Phi_F$) plot of **CAS-C1** aggregate (10 mM Tris, 100 mM K^+, pH 7.2).

ECD Spectra

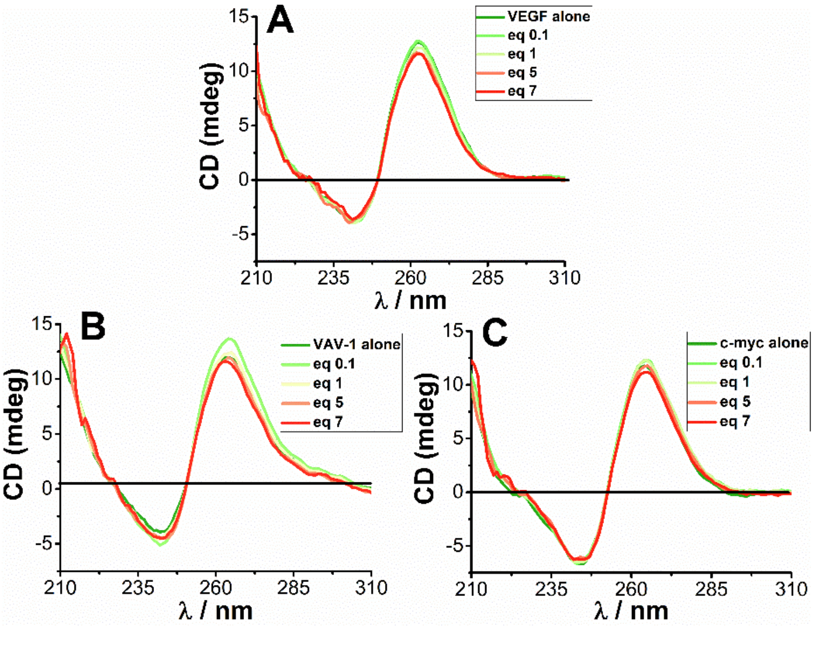

Figure S27 Electronic circular dichroism (ECD) spectra of A) VEGF, B) VAV-1 and C) c-myc in buffered water solution (3 × 10^{-6} M, TRIS buffer c = 10 mM, pH = 7.2, KCl c = 100 mM) at 25 °C in the absence and presence of **CAS-C1** at different ratios.

6. NMR and HRMS spectra of CAS-C1

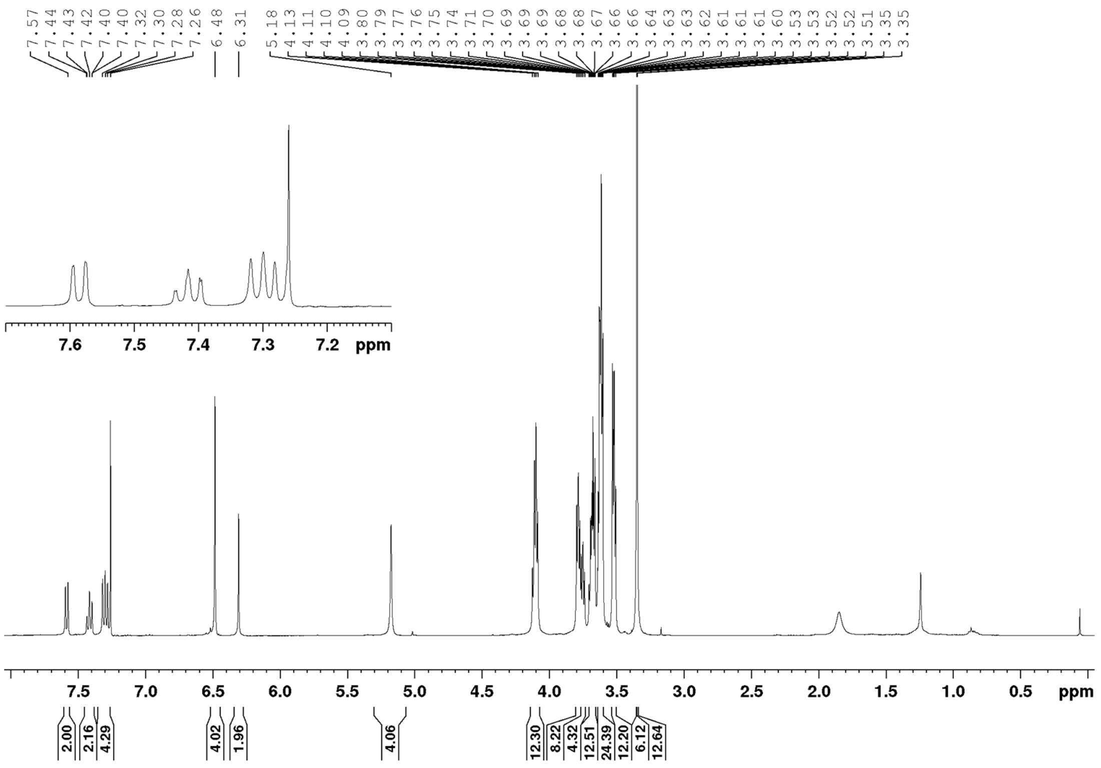

Figure S28 ^{1}H NMR (400 MHz, 298 K) spectrum of **CAS-C1** in $CDCl_3$ (Inset: Signals of aromatic protons).

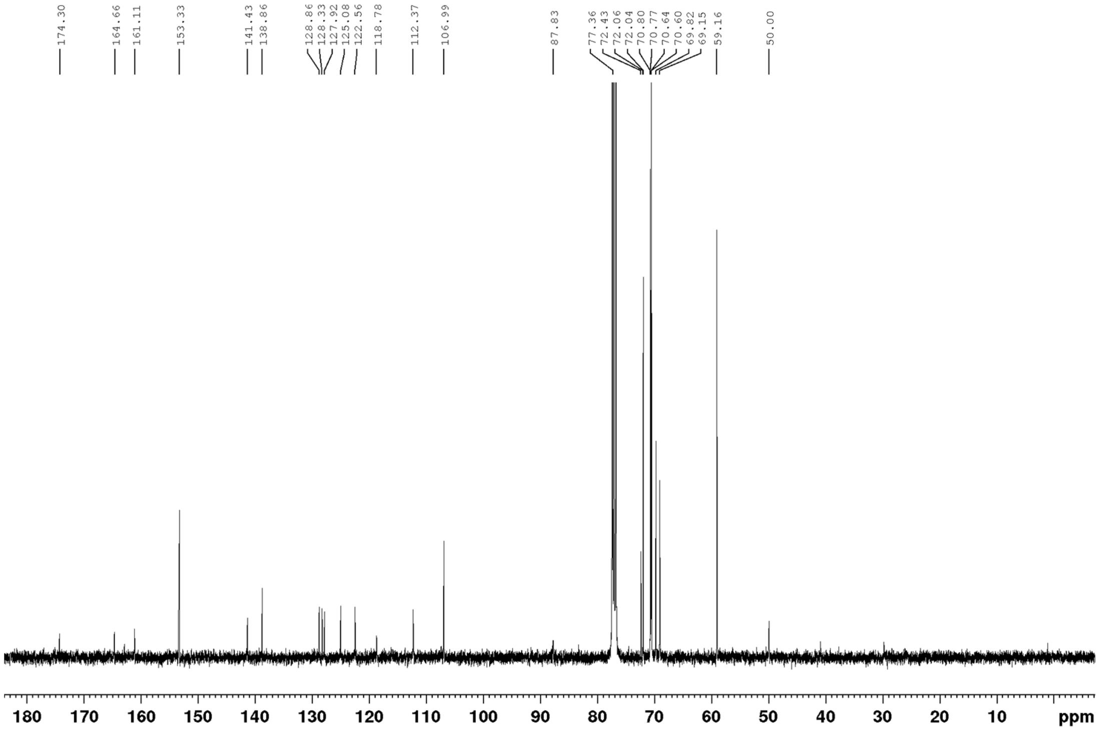

Figure S29 ^{13}C NMR (100 MHz, 298 K) spectrum of **CAS-C1** in $CDCl_3$.

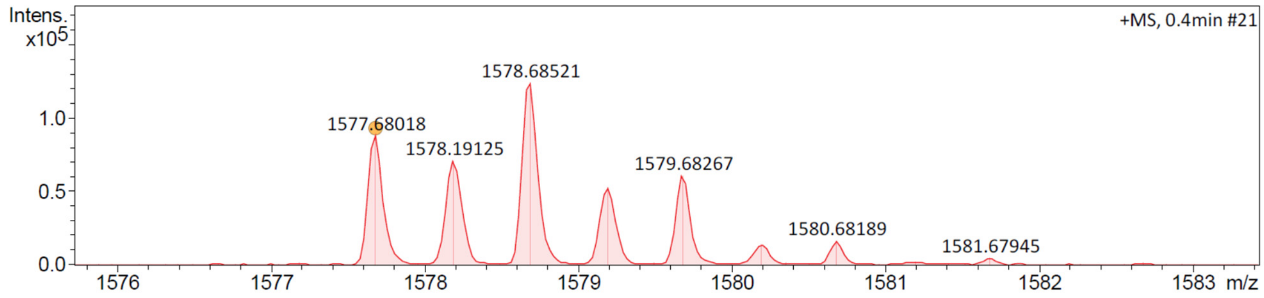

Figure S30 HRMS-ESI spectrum of **CAS-C1** (positive mode, acetonitrile/chloroform).

7. References

1 A. Alessi, M. Salvalaggio and G. Ruzzon, *J. Lumin.*, 2013, **134**, 385-389.
2 J. R. Lakowicz, *Principles of Fluorescence Spectroscopy*, Kluwer Academic / Plenum Publishers, 1999.
3 C. Würth, M. Grabolle, J. Pauli, M. Spieles and U. Resch-Genger, *Nat. Protoc.*, 2013, **8**, 1535-1550.
4 E. Largy and J.-L. Mergny, *Nucleic Acids Res.*, 2014, **42**, e149.
5 A. Kerkour, J. Marquevielle, S. Ivashchenko, L. A. Yatsunyk, J.-L. Mergny and G. F. Salgado, *J. Biol. Chem.*, 2017, **292**, 8082-8091.
6 J. M. Nicoludis, S. P. Barrett, J.-l. Mergny and L. A. Yatsunyk, *Nucleic Acids Res.*, 2012, **40**, 5432-5447.
7 A. De Rache and J.-L. Mergny, *Biochimie*, 2015, **115**, 194-202.
8 N. Kumar and S. Maiti, *Nucleic Acids Res.*, 2008, **36**, 5610-5622.
9 A. T. Phan, *FEBS J.*, 2010, **277**, 1107-1117.
10 A. T. Phan, V. Kuryavyi, S. Burge, S. Neidle and D. J. Patel, *J. Am. Chem. Soc.*, 2007, **129**, 4386-4392.
11 Z. Zhang, J. Dai, E. Veliath, R. A. Jones and D. Yang, *Nucleic Acids Res.*, 2010, **38**, 1009-1021.
12 N. S. Makarov, M. Drobizhev and A. Rebane, *Opt. Express*, 2008, **16**, 4029-4047.
13 M. Y. Berezin, C. Zhan, H. Lee, C. Joo, W. J. Akers, S. Yazdanfar and S. Achilefu, *J. Phys. Chem. B*, 2011, **115**, 11530-11535.
14 P. Thordarson, *Chem. Soc. Rev.*, 2011, **40**, 1305-1323.
15 D. B. Hibbert and P. Thordarson, *Chem. Commun.*, 2016, **52**, 12792-12805.
16 M. J. Frisch, G. W. Trucks, H. B. Schlegel, G. E. Scuseria, M. A. Robb, J. R. Cheeseman, G. Scalmani, V. Barone, B. Mennucci, G. A. Petersson, H. Nakatsuji, M. Caricato, X. Li, H. P. Hratchian, A. F. Izmaylov, J. Bloino, G. Zheng, J. L. Sonnenberg, M. Hada, M. Ehara, K. Toyota, R. Fukuda, J. Hasegawa, M. Ishida, T. Nakajima, Y. Honda, O. Kitao, H. Nakai, T. Vreven, J. A. Montgomery, J. E. Peralta, F. Ogliaro, M. Bearpark, J. J. Heyd, E. Brothers, K. N. Kudin, V. N. Staroverov, R. Kobayashi, J. Normand, K. Raghavachari, A. Rendell, J. C. Burant, S. S. Iyengar, J. Tomasi, M. Cossi, N. Rega, J. M. Millam, M. Klene, J. E. Knox, J. B. Cross, V. Bakken, C. Adamo, J. Jaramillo, R. Gomperts, R. E. Stratmann, O. Yazyev, A. J. Austin, R. Cammi, C. Pomelli, J. W. Ochterski, R. L. Martin, K. Morokuma, V. G. Zakrzewski, G. A. Voth, P. Salvador, J. J. Dannenberg, S. Dapprich, A. D. Daniels, O. Farkas, J. B. Foresman, J. V. Ortiz, J. Cioslowski and D. J. Fox, *Gaussian 09*, Gaussian, Inc., 2009.
17 RCSB Protein Data Bank, https://www.rcsb.org/, (accessed April, 2018).
18 O. Trott and A. J. Olson, *J. Comput. Chem.*, 2010, **31**, 455-461.
19 E. F. Pettersen, T. D. Goddard, C. C. Huang, G. S. Couch, D. M. Greenblatt, E. C. Meng and T. E. Ferrin, *J. Comput. Chem.*, 2004, **25**, 1605-1612.
20 M. Su, S.-Y. Shi, Q. Wang, N. Liu, J. Yin, C. Liu, Y. Ding and Z.-Q. Wu, *Polym. Chem.*, 2015, **6**, 6519-6528.
21 U. Mayerhöffer, M. Gsänger, M. Stolte, B. Fimmel and F. Würthner, *Chem. Eur. J.*, 2013, **19**, 218-232.
22 M. M. J. Smulders, M. M. L. Nieuwenhuizen, T. F. A. de Greef, P. van der Schoot, A. P. H. J. Schenning and E. W. Meijer, *Chem. Eur. J.*, 2010, **16**, 362-367.
